# Review of EEG, ERP, and Brain Connectivity Estimators as Predictive Biomarkers of Social Anxiety Disorder

**DOI:** 10.3389/fpsyg.2020.00730

**Published:** 2020-05-19

**Authors:** Abdulhakim Al-Ezzi, Nidal Kamel, Ibrahima Faye, Esther Gunaseli

**Affiliations:** ^1^Centre for Intelligent Signal and Imaging Research, Department of Electrical and Electronic Engineering, Universiti Teknologi PETRONAS, Seri Iskandar, Malaysia; ^2^Psychiatry Discipline Sub Unit, Universiti Kuala Lumpur, Ipoh, Malaysia

**Keywords:** electrocortical endophenotypes, neurofeedback, cross-frequency coupling, delta-beta correlation, effective connectivity, event-related potential, visual-event potential

## Abstract

Social anxiety disorder (SAD) is characterized by a fear of negative evaluation, negative self-belief and extreme avoidance of social situations. These recurrent symptoms are thought to maintain the severity and substantial impairment in social and cognitive thoughts. SAD is associated with a disruption in neuronal networks implicated in emotional regulation, perceptual stimulus functions, and emotion processing, suggesting a network system to delineate the electrocortical endophenotypes of SAD. This paper seeks to provide a comprehensive review of the most frequently studied electroencephalographic (EEG) spectral coupling, event-related potential (ERP), visual-event potential (VEP), and other connectivity estimators in social anxiety during rest, anticipation, stimulus processing, and recovery states. A search on Web of Science provided 97 studies that document electrocortical biomarkers and relevant constructs pertaining to individuals with SAD. This study aims to identify SAD neuronal biomarkers and provide insight into the differences in these biomarkers based on EEG, ERPs, VEP, and brain connectivity networks in SAD patients and healthy controls (HC). Furthermore, we proposed recommendations to improve methods of delineating the electrocortical endophenotypes of SAD, e.g., a fusion of EEG with other modalities such as functional magnetic resonance imaging (fMRI) and magnetoencephalograms (MEG), to realize better effectiveness than EEG alone, in order to ultimately evolve the treatment selection process, and to review the possibility of using electrocortical measures in the early diagnosis and endophenotype examination of SAD.

## Introduction

The diagnosis of social anxiety disorder (SAD or; social phobia) was first introduced in the Third Edition of the Diagnostic and Statistical Manual of Mental Disorders (DSM-III) in 1980 (Nathan, 2019). SAD is defined as a distinct and persistent fear of one or more social situations in which a person is exposed to unfamiliar people or possible scrutiny by others (Radtke et al., 2020). People with SAD suffer anxiety from the potential risk of embarrassment or humiliation due to inadequate social performance (Langer et al., 2019). Consequently, they often avoid social situations, and when the situation is unavoidable, they experience severe anxiety and stress. SAD is associated with an increased risk of developing comorbid conditions, especially major depression, acute grief, and substance abuse disorders (Rapee and Spence, 2004). Basically, SAD manifests early in life and portends significant social functional impairment, psychiatric comorbidity, and persistent emotional, cognitive, and behavioral disabilities (Grant et al., 2005). SAD can cause serious damage to the lives of those who suffer from it. When SAD symptoms disrupt an individual’s daily life activities by reaching the level of avoidance of social situations (Givon-Benjio and Okon-Singer, 2020), these individuals usually meet the diagnostic standards for SAD (Welander-Vatn et al., 2019). For example, an individual may reject a job opportunity that requires frequent interaction with new people or may avoid going out. SAD interferes with important aspects of daily life, such as academic and professional environments, family relationships, and social activities (Frandsen et al., 2019). Social anxiety typically remains highly stable and persistent across the lifespan if left untreated (Yonkers et al., 2001). SAD affects approximately 15 million American adults (6.8% of the population) and is the second most common psychiatric condition after specific phobia (8.7%) (Kessler et al., 2005). Current surveys estimate that the lifetime prevalence of social phobia in Western countries is about 7–13%. However, prevalence percentages vary vastly and are liable to errors due to some methodological factors such as diagnostic criteria, diagnostic thresholds, and evaluation methods (Furmark, 2002). Some countries have investigated the population ratio of children and adolescents with SAD among the general population and college students. Incidence rates of SAD are estimated to be between 13 and 16% (Furmark, 2002; Baptista et al., 2012). As with adult studies, several methods have been applied to investigate the wide variation in SAD prevalence estimation. A large New Zealand statistical study had declared that 11.1% of 18-year-olds fulfilled the criteria for SAD (Baptista et al., 2012). Nonetheless, a large British epidemiological statistic (Ford et al., 2003) reported that only 0.32% of children aged 5–15 years had this disorder, which is higher than the incidence rates of other mental illnesses, such as post-traumatic stress disorder (PTSD) and obsessive-compulsive disorder (OCD), but lower than that of panic disorder, specific phobia, and generalized anxiety. This study found that the diagnostic rate of SAD increases slightly with age in men more than in women. A large US Child and Adolescent Psychiatric Assessment study reported that 4.1% of the participating children between the ages of 9 and 13 years were diagnosed with SAD (Costello et al., 2003), while a German study estimated the incidence of SAD is 4% in children between the ages of 14 and 17 years (Iffland et al., 2014). The most serious form of SAD can paralyze patients emotionally and physically, and many people struggle to cope with other psychological challenges such as internal sensation novelty seeking. For example, a study found that about half the population of people with SAD have comorbid mental illnesses, drug addiction or alcohol problems (Robichaud et al., 2019). However, most of the available data on SAD epidemiology originate from high-income countries in the West. European epidemiological data is highly correlated with US data, confirming the high prevalence, comorbidity, and morbidity of SAD (Perna et al., 2020).

Furthermore, medically ill individuals with SAD are at a higher risk of a chronic course of the illness or less complete recovery. Consequently, SAD patients receive special medical care, e.g., antidepressant medications that alleviate depression and anxiety symptoms (Hansen et al., 2008; Potter, 2019). Cognitive-behavioral therapy (CBT) and other psychotherapies are believed to improve positive emotions and create balanced neuronal mindfulness. Generally, CBT plays a crucial role in sustaining interpersonal sensation. Shreds of evidence from clinical interventions indicate that CBT plays a fundamental role in managing SAD (Morrison et al., 2019).

Based on the research objectives of this study, the key phrase is ‘social anxiety disorder.’ We then specify the alternatives of the keyword (‘social anxiety disorder’) social phobia, delta-beta correlation, frontal alpha asymmetry, functional connectivity, event-related potential, visual-event potential, and effective connectivity. After the search terms have been specified, they are assembled into a search series that is applied in the research process. In this review, the AND operator is used to connect the different search terms into a single search chain. The following online databases serve as the resources that were searched: Frontiers, IEEE Xplore, SpringerLink, BioMed Central, Wiley Interscience, ACM, and ScienceDirect. We also manually checked the reference list of the corresponding primary studies to ensure that our review is complete. The selection of articles was conducted by applying a set of inclusion and exclusion criteria. The exclusion criteria are: articles without any of the listed keywords, articles not in the English language, incomplete articles, short articles, replicated articles, and all studies irrelevant to the enquiry. The Inclusion criteria were set based on the following: articles with listed keywords, English studies, and full-text papers. Based on the inclusion and exclusion statement, we excluded 93 studies out of 253 primary studies. We gathered a final 160 studies from the primary and secondary database searches to report in this study.

The main objective of this review is to provide an extensive overview of the most frequently studied EEG, ERP, and brain connectivity measures during rest, anticipation, visual stimulus tasks, and recovery from performing social tasks. However, we found it essential to include functional magnetic resonance imaging (fMRI) and magnetic resonance imaging (MRI) studies to validate the findings of the EEG studies. Furthermore, we excluded positron emission tomography (PET) scan based studies because the PET scans use a radioactive tracer, which the patient receives via injection. However, PET is not an accurate model for determining neuronal locations (Lefevre et al., 2019).

## Cognitive Models of Social Anxiety Disorder

**FIGURE 1:**
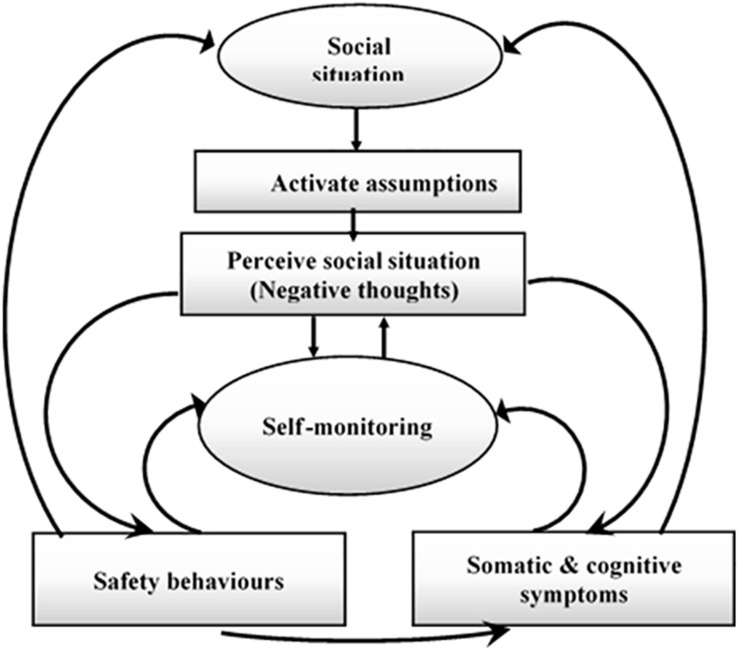
A cognitive model of SAD (Clark and Wells, 1995).

Contemporary cognitive models of SAD (Clark and Wells, 1995; Hofmann, 2007), and (Rapee and Heimberg, 1997) have been widely used to extend the theoretical framework for understanding the key emotional maintenance processes involved in SAD (Arditte Hall et al., 2019). Explicitly, these frameworks suggest that negative beliefs, biased rumination over social cues, and evasion of anxiety stimulators contribute to the expansion and maintenance of SAD. Prior reported models (Arditte Hall et al., 2019) are theoretically identical and primarily focus on the maintenance attributes experienced by SAD patients. Furthermore, study by Hofmann (2007) spotlights the main role of perceived high social standards that trigger anticipatory anxiety before social activities. Based on the cognitive model proposed in a previous study (Clark and McManus, 2002), patients with SAD are more likely to view and apprehend social information as threats because of their cognitive bias about themselves, others, and the surroundings. For instance, patients with SAD typically ascribe extremely high criteria for social life, which naturally induces a perception of failure and anxiety (Clark and Wells, 1995). As illustrated in Figure 1, Clarke’s model assumes that there are many different processes involved in maintaining SAD. Individuals with SAD encountering social situations are more likely to recall destructive assumptions of social threats, resulting in a shift to self-attention and exhaustive self-monitoring. The unsociable and self-focused behaviors further elevate the activation of neurophysiological and physical attributes of anxiety, such as trembling, sweating, and rumination over negative beliefs. Patients with SAD then use these physical signal and destructive assumptions to create negative psychological representations of themselves (because they think they will be criticized and judged negatively by others). Moreover, patients with SAD are familiar with being involved in safe behaviors (Clark and McManus, 2002; Kim, 2012) such as avoidance or internal psychological thoughts (e.g., rehearsing what to say or do next; Reyes et al., 2020). This model assumes that social apprehension is linked with impracticable social standards and insufficiency in selecting attainable social goals. When experiencing and confronting challenging social events, people with SAD shift their attentiveness and attention toward their anxiety, view themselves negatively as a social object, overestimate the negative consequences of a social confrontation, imagine that they have no control over their emotional responses, and view their social skills as inadequate for effectively facing the social situation (Hofmann, 2007). The cognitive models indicate that, when confronted with a social threat, individuals with SAD focus their attention internally and engage in a process of detailed self-observation (Hofmann, 2000; Hirsch et al., 2003). The model illustrated in Figure 1 is contingent on the idea that various repeated behaviors and thought patterns are associated with the somatic and emotional indicators of social anxiety. The main cognitive models of SAD (Clark and Wells, 1995; Hofmann, 2007) and (Reyes et al., 2020) are theoretically structured and highlight the importance of negative beliefs subsequent to social events as a modulating factor in the SAD cycle. The practice of negative rumination arises in patients with SAD when they indulge in an extreme mental assessment of their past social performance (Brozovich and Heimberg, 2008). Researchers have found that individuals with SAD engage in excessive and persistent negative reflections before and after social assessment events (Rachman et al., 2000; Vassilopoulos et al., 2014). Individuals with severe social anxiety will have a greater degree of negative rumination after social events (e.g., improvised speech) (Edwards et al., 2003) or any social activity that requires interactions with others (Mellings and Alden, 2000). A previous study (Vassilopoulos et al., 2014) performed one of the first empirical elucidations of the relationship between trait anxiety and higher levels of negative anticipation, in which individuals with SAD reported that their recurrent negative thoughts about future social events were intrusive, uncontrollable, and interfered with their ability to concentrate during social events. While individuals, with high social anxiety (HSA) and low social anxiety (LSA) engage in predicting the cost and consequences of future social events, these predictions are significantly more negative among participants HSA. When undergraduate students with LSA and HSA were asked to participate in tasks causing social anxiety and pre-event rumination prior to short presentation, HSA participants reported more anxiety and a more negative self-image than LSA participants (Brown and Stopa, 2007; Blöte et al., 2019).

A previous study (Coles et al., 2002) investigated post-event processing by assessing the perspectives of memory in patients with SAD involved in delivering a speech and carrying out social interaction. As predicted, participants with severe SAD recalled the social situation more frequently than those with low anxiety some days later. Many researchers (Edwards et al., 2003; Abbott and Rapee, 2004) and (Perini et al., 2006) have found support for their hypothesis that individuals with a higher level of social anxiety recalled more negative responses than positive responses during treatment sessions, while healthy controls (HCs) do not exhibit any bias in memory. Regarding post-processing, HSA individuals expressed dissatisfaction with the negative factors of improvised speech and negative feedback at 1-week intervals, while the HCs did not exhibit this inclination. Individuals with SAD experience more negative ideations under post-rumination conditions and create more counterfactual reasoning and beliefs. Consequently, patients with severe SAD have generated more negative “hypothetical” statements after reading about a mildly social situation (Mellings and Alden, 2000; Rachman et al., 2000). Post-event treatment has been applied to investigate the memory reactivations in individuals with SAD and HCs who rated events negatively. The results showed that people with SAD recalled significantly more negative and embarrassing memories than HCs, regardless of the type of post-treatment (Field et al., 2004). Overall, these findings confirm that people with SAD assess their reactions to social events in a negative light and indulge in ongoing negative thinking. These features reveal encouraging indications that these cognitive impairments of biases can be alleviated.

## Biomarkers of Social Anxiety Disorder

Various studies have critically discussed the empirical evidence on information-processing in SAD and utilize several social cognitive tasks to investigate the electrophysiological effects and the associated attention in response to these tasks (Davidson et al., 2000; Miskovic et al., 2010, 2011a,b). These studies aimed to reveal the association between electrophysiological and trans-diagnostic symptom dimensions associated with SAD. The results presented (MacNamara et al., 2013) show a prominent correlation between the dimensions of neurological responsiveness and the dimensions of anxiety symptoms that exceed traditional diagnostic boundaries established via performing working memory tasks. Based on these results, a recent study (Yeung and Fernandes, 2019) investigated whether SAD is associated with a memory bias for threat or whether the memory bias is created by threatening or neutral distractors. For threats and neutral targets, there are no differences in memory between the study groups. However, awareness of social threats is more significantly enhanced and recognized by patients with severe SAD than those with mild SAD. Using latent class growth curve analysis, it was proven that salivary cortisol levels in different time assessments are stronger in children with high, stable salivary cortisol. The hypothalamic-pituitary-adrenal (HPA) axis is a network combination of direct impacts and feedback connections between three elements: the hypothalamus, pituitary gland, and adrenal glands. These three elements and their communication create the HPA axis, a significant mechanism of the neuroendocrine pathways that regulate stress reactions and regulate several processes in the body, including emotions (Malenk et al., 2009). An alternate interpretation is that SAD is a reaction to a tension-related situation, with the core endocrine reaction seen in humans being a response to stress activating the HPA axis which leads to an increase in cortisol (Condren et al., 2002). The HPA axis is associated with cortisol production, and higher levels of cortisol is associated with anxiety, fear, and avoidance (Poole and Schmidt, 2019). Acute stressors (e.g., anxiety) can alter many biological functions, such as those of the HPA axis (Foley and Kirschbaum, 2010), the body’s defense system (Steptoe et al., 2007), the regulation process of the autonomic nervous system (Xhyheri et al., 2012), HPA axis activity (Juruena et al., 2020) and the enteric nervous system (Ziegler, 2012). Psychologically, acute anxiety is a subjective negative experience that can have positive and negative effects on cognition (Starcke and Brand, 2012). Consistent with these findings, heart rate variability (HRV) was examined in patients with SAD and HCs at a baseline with anxiety as a stressor. The small effect size of SAD supports a more modest reduction in HRV in individuals with SAD (Pittig et al., 2013). HRV during a resting state and while performing social cognitive tasks was not significant in participants with SAD (Harrewijn et al., 2018a). In comparison, SAD was negatively linked to HRV during an emotional recognition task, and participants exhibited a significant decrease in HRV (Madison, 2019). Reduction in HRV was found in females with SAD as opposed to HCs and in patients on psychotropic medication as opposed to patients without medication (Alvares et al., 2013).

**FIGURE 2:**
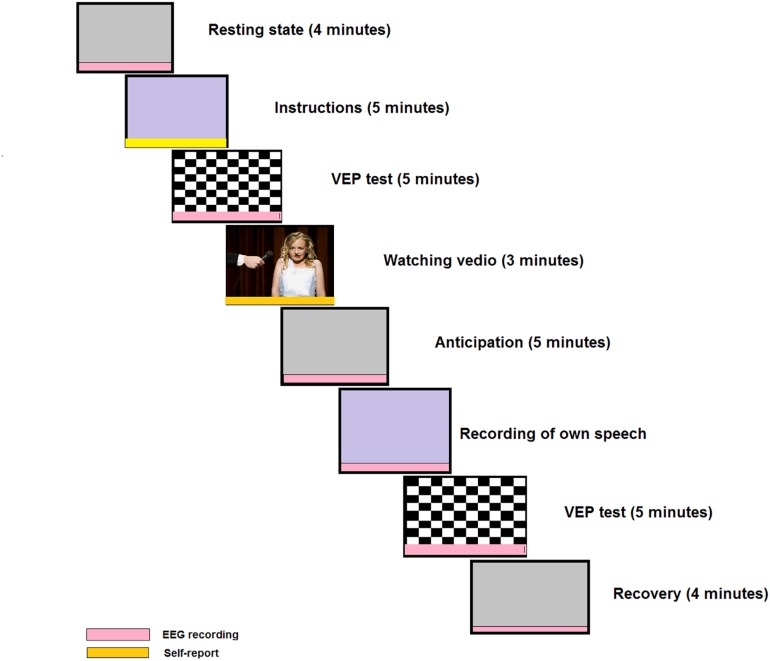
Example of a social cognitive task. This task includes visual evoked potentials (VEPs) test before and after self-presentation; and this design is novel compared to the more frequently used designs. The red color indicates EEG recording sessions and the yellow color indicates self-assessment. A modified version of the original in the journal of Cognitive, Affective and Behavioral Neuroscience, 16, Harrewijn A., Van der Molen M. J., Westenberg P. M., Putative EEG measures of social anxiety: Comparing frontal alpha asymmetry and delta-beta cross-frequency correlation, 1086–1098-Copyright (2016), with permission from Elsevier.

We designed a novel social performance task, shown in Figure 2, based on a previous experimental design in Rinck et al. (2013) and Harrewijn et al. (2016), in which all subjects are given a self-presentation task to be delivered in front of an audience and the presentation is videotaped, unlike a previous study (Miskovic et al., 2010). The EEG signals are recorded in six stages: resting-state, anticipation, speech, VEPs, and recovery from social task. During three stages of the social performance task (Figure 2), participants are asked to indicate their feeling on how much they want to engage in the next stage of the experiment with a rating 0 (NO) to 10 (YES) to measure avoidance. We are the first to investigate the severity of SAD using VEPs test and the analysis of effective connectivity through the whole social task as shown in Figure 2.

## Electrophysiological Correlation of Social Anxiety Disorder

Different studies have been conducted to determine the electroencephalographic and electrophysiological correlation of anticipatory anxiety induced in individuals with social phobia. There are many modalities for capturing information on the structure and functions of the brain. The three commonly and frequently used modalities are MRI, magnetoencephalography MEG and electroencephalography EEG. Among these methods, EEG is the most versatile and most cost-effective method for studying brain activity, and is an effective model for studying the neural correlates of social anxiety and for obtaining large-scale connectivity model of brain functions. Over the last decade, the analysis of electrophysiological signals has been continuously popularized by using blind source separation (BSS) techniques used for dimensional reduction. BSS is also used to separate the information in mixtures of signals into multivariate recorded data channels to prepare multivariate data sets for more general data analysis and to extract the biomarkers of SAD under different conditions for EEG data. The following subsections outline the most prominent EEG-based biomarkers of social anxiety.

### Delta-Beta Cross-Frequency Correlation in Social Anxiety

EEG activity reflects the temporal aggregation of synchronous activity of millions of spatially aligned cortical neurons. The most deliberated waveforms include delta (0.5–4 Hz), theta (4–7 Hz), alpha (8–12 Hz), sigma (12–16 Hz), and beta (13–30 Hz). Delta-beta cross-frequency correlation is a widely used measure in the investigation of social anxiety (Table 1). This measure represents the cross-frequency correlation between the amplitude of delta-band and beta-band oscillations and is known as a delta-beta correlation. The spectral coupling between the delta and beta oscillations has been proven to be related to social anxiety (Davidson et al., 2000; Miskovic et al., 2010, 2011a,b). Broadly, EEG bands are associated with different functional and behavioral correlations. For instance, a slow-wave (SW) brain oscillation like delta is associated with the subcortical regions responsible for motivation, mood, and reward processing (Knyazev, 2012; Newson and Thiagarajan, 2019). In contrast, a fast brain wave reflects intercortical connections and is activated when attentional control, cognitive processing, and regulation is required (Engel et al., 2001). In general, beta waves exist when a person is vigilant, attentive, involved in problem-solving, judgment, decision making, or mental focus. It is believed that the correlation between the fast-wave (FW) and SW frequency of the frontal lobe reflects an interaction between the cortical and subcortical circuits (Knyazev et al., 2019; De Pascalis et al., 2020) and (Knyazev, 2012). Thus, the synchronized increment in SW and FW activity in anxiety-related behavioral profiles is thought to reflect neural communications between two different brain regions. A series of adult studies found that the magnitude of the frontal delta-beta coupling is sensitive to steroidal hormones, including cortisol. A recent study (Poole and Schmidt, 2019) broadened an electrophysiological research to children aged 7–17 years and found that the frontal EEG delta-beta rhythm is strong in children with relatively high levels of basal cortisol and SAD. These findings suggest that longitudinal patterns of neuroendocrine stress activity and social anxiety may be linked with EEG power in slow and fast frontal cerebral oscillations during early childhood. Previous studies discovered that frontal delta-beta coupling is stronger among young adults who scored higher inherited cortisol levels (Engel et al., 2001), and that it is further strengthened after exogenous cortisol administration in adults (Knyazev, 2007). These results demonstrate that cortisol levels may be related to the increment of SW and FW activity in the frontal lobe. Although behavioral studies in the context of delta-beta correlation are limited, some studies have found stronger delta-beta coupling in relation to the severity of social panic (Knyazev and Slobodskaya, 2003) and dysregulated fear (Schutter and van Honk, 2005) among preschool children and infants with high cortisol reactivity (van Peer et al., 2008). An intergenerational study found that children with socially anxious parents exhibit a more grounded resting-state frontal delta-beta coupling than those with healthy parents in response to social stressors, which might be a possible endophenotype of SAD (Brooker et al., 2016; Harrewijn et al., 2018c). Greater social fears are associated with greater positive frontal coupling between delta and beta bands.

**TABLE 1:** Summary of works about delta-beta correlation related to SAD.

Delta-beta correlation	Study	Participants	Task	EEG main findings
Resting state (RS)	Edwards et al., 2003	HSA vs. LSA	RS Cortisol vs. placebo administration	Greater positive delta-beta correlates. Greater Delta-beta correlates after both groups had administrated by cortisol
	Miskovic et al., 2010	High vs.low adult	RS	Greater positive delta-beta correlation. Greater positive delta-beta correlates.
	Poole and Schmidt, 2019	High and low SAD school-age children	High and low cortisol	
	Najjar and Brooker, 2017	HSA vs. LSA children	RS	Stronger prefrontal delta-beta correlation
	Harrewijn et al., 2016	HSA vs. LSA	RS	No significant difference
	Miskovic et al., 2010	HSA vs. LSA	RS	No significant difference
	Phelps et al., 2016	HAS children	RS	Stronger delta-beta correlation in trait anxiety.
	Schutter and van Honk, 2004	HSA vs. LSA	RS	Significant decrease in delta-beta
	van Peer et al., 2008	HSA vs. LSA	Steroid hormone	Increase in frontal delta-beta coupling
	Knyazev and Slobodskaya, 2003	High and low SAD school-age children	Cortisol administration	Greater coupling at frontal electrodes
	Knyazev et al., 2006	HSA vs. LSA	RS	HSA Increasein frontal delta-beta coupling
	De Pascalis et al., 2020	HSA vs. LSA	RS	A significant positive delta-beta correlation in HAS
	Knyazev et al., 2019	MDD/SAD vs. HC	RS	Associated with an enhancement of cross frequency
Mutual: resting anticipation recovery	Coles et al., 2002	SAD vs. control Pre-therapy 1 Pre-therapy 2 Mid-therapy 3 Post-therapy 4	RS Anticipation	Lower positive delta-beta correlates in session 1 to session 2 and from session 1 to session 4 Lower positive delta-beta correlates in pretreatment to post-treatment.
	Harrewijn et al., 2016	HSA vs. LSA	RS Anticipation	No significant difference Greater negative delta-beta correlation for frontal electrodes.
	Brown and Stopa, 2007	HSA vs. LSA	Recovery	Greater negative delta-beta correlation in frontal lobe.
	Engel et al., 2001	HSA vs. LSA	RS Anticipation	No significant difference. Greater positive delta-beta correlation (F4)
	Knyazev and Slobodskaya, 2003	HSA vs. LSA	RS Anticipation	No significant difference Correlation was higher in LSA than HSA participants during the early anticipation
	van Peer et al., 2008	Preschool children	Socialtask	Stronger delta-beta coupling in relation to high levels of observed social fear
	Brooker et al., 2016	HSA vs. LSA	RS Anticipation	No significant difference. Negative delta-beta coupling
	Harrewijn et al., 2018b	HSA vs. LSA	Recovery	No significant difference. Negative delta-beta coupling
	Bishop, 2007	SAD and GAD	Anticipation	
	Morillas-Romero et al., 2015	HSA and LSA	Anticipation	HSA have lower significant amplitude-amplitude correlation than LSA
	Poole et al., 2020	HSA and LSA	Anticipation	Increase in central and parietal delta-beta coupling in HAS
	Knyazev et al., 2019	HSA vs. LSA (adult vs. children)	Recovery	Increase in parietal delta-beta coupling in children
	Poppelaars et al., 2018	Female HSA vs. LSA	Recovery	Higher delta-beta coupling in HSA females
	Brozovich and Heimberg, 2008	HSA vs. LSA	Recovery	Negative delta-beta correlation in LSA

#### Delta-Beta Correlation in Resting State (Baseline)

EEG signals were recorded in preschool children at rest, and these were used to test whether the individual differences in delta-beta cross frequency are related to sympathetic or cruel parental behaviors (Knyazev and Slobodskaya, 2003; Poole and Schmidt, 2019). Several pieces of evidence suggest a greater coupling at the frontal electrodes in school-age children whose fathers exhibit high levels of ruthless parental behaviors than in those whose fathers exhibit low levels of harshness. Delta-beta coupling is a real-time indicator of cortical neural network down-regulation of mood-based responsiveness in subcortical networks (Najjar and Brooker, 2017). Children with dysregulated fright or high avoidance in low-threat circumstances showed a higher correlation in the baseline state than those who exhibited low levels of avoidance in low-threat environments (Phelps et al., 2016). Although the investigation of delta-beta coupling in preschool-aged children is still in the early stages, preliminary findings suggest that resting-state delta-beta coupling may be indicative of trait-level tendencies in cognitively oriented neural systems to down-regulate neural systems for emotional reaction (Phelps et al., 2016). An attempt was made to examine the relationship between delta-beta EEG spectral coupling and endogenous testosterone levels in men in a resting regional brain activity state. Men with high testosterone levels showed non-significant delta-beta coupling (delta-beta decoupling), while men with shrinking testosterone levels showed significant delta-beta coupling. These relationships are only constructed in the frontal lobe (Miskovic and Schmidt, 2009). The level of delta-beta coupling can be influenced and manipulated experimentally. For instance, the use of synthetic cortisol (van Peer et al., 2008) and anxiety induction (Knyazev et al., 2006) results in an increase in frontal delta-beta coupling. In contrast, subcutaneous administration of testosterone resulted in a significant decrease in delta-beta cross-frequency, which is in line with the anxiolytic attributes of the steroid hormone (Schutter and van Honk, 2004). Several findings regarding cross-frequency coupling at baseline have been reported, with a previous study (Miskovic et al., 2011b) proving that the correlation in SW-FW before psychotherapy is more enhanced than after treatment for SAD. Nonetheless, pre-treatment delta-beta correlation in patients with SAD shows no significant changes compared to HCs (Miskovic et al., 2011b), and the cross-frequency coupling between fast and slow bands is greater than that at the low naturally inhibited group (van Peer et al., 2008). Conversely, two studies did not find any difference between trait and state social anxiety (Miskovic et al., 2010; Harrewijn et al., 2016). It was hypothesized that the magnitude of the power between delta and beta oscillation in spontaneous EEG is related to the level of hormones such as cortisol and testosterone; therefore, studying social anxiety during resting state is a good idea to be investigated. A previous study (Miskovic et al., 2010) found evidence suggesting that the level of frontal spectral coupling between SW and FW can be used to distinguish socially anxious individuals and to quantify the severity of SAD during social interactions. Specifically, adults with HSA showed significantly greater delta-beta coupling of the right frontal electrodes (right hemisphere) than adults with LSA in resting state (De Pascalis et al., 2020).

#### Delta-Beta Coupling in the Anticipation State

A very recent study (Poole and Schmidt, 2019) found that preschool children who have unsteady and temperamental antecedents of SAD exhibited relatively high frontal delta-beta correlation, as reported in Knyazev et al. (2019) and Poole et al. (2020). It is obvious that although the overall coupling in frontal cross-frequency in children with SAD in this study is stronger, this pattern seems to be controlled primarily by the right frontal lobe when examining the separate hemispherical coupling. The results presented in this study are identical to those observed previously, with the HSA participants showing lower significant amplitude-amplitude correlation during a social performance task than the LSA participants who displayed significant values during early anticipation and under all conditions (Morillas-Romero et al., 2015). Delta-beta coupling as an electrocortical measure of SAD is believed to be more promising when socially anxious participants anticipate social stress. Compared to LSA individuals, the HSA group shows an increase in positive delta-beta correlation during the anticipation period prior to cognitive therapy. The increment in positive cross-frequency coupling between SW and FW during the anticipation period in patients with SAD is reduced after CBT and there is no dissimilarity between individuals with high or low SAD (Miskovic et al., 2011a). Patients with SAD shows a higher positive delta-beta correlation during the anticipation period than individuals with lower SAD (Miskovic et al., 2010). In contrast, a greater negative cross-talk between delta waves and beta waves has been reported in patients with SAD than in those with low SAD (Harrewijn et al., 2016). In addition, a higher right frontal brain power was found in HSA but not in normal controls when anticipating self-presentation. It is believed that negative SW-FW coupling might be explained by an increase in the connection between the internal (e.g., amygdala and insula) and external (e.g., cortex) sites in the brain in opposite directions. A known imbalance between the subcortical and cortical networks in patients with generalized social anxiety disorder (GAD) (Bishop, 2007) and SAD (Miskovic and Schmidt, 2012), may result in a negative delta-beta coupling. Contemporary electrocortical indices indicate that the mid-frontal theta (4–8 Hz) oscillation in the electroencephalogram provides new insights into the processing of social repudiation by the brain (Knyazev et al., 2019). These findings demonstrated that mid-frontal theta (4–8 Hz) oscillation is very responsive to social repudiation but only when peer repudiation is unpredicted, which indicates that the frontal theta is controlled by a widely different neural network implicated in saliency perception and conflict detection (van der Molen et al., 2017). The correlation between delta and beta has been proven to be very sentient to external influential factors because it differentiates between perfect and thoughtless performance conditions (Miskovic et al., 2011b).

#### Delta-Beta Coupling in the Recovery State

Although post-processing is a very crucial aspect of social anxiety, few studies have investigated delta-beta coupling after predicting social stress conditions (recovery). In a study by Harrewijn et al. (2016), the individuals with higher SAD show an increased negative delta-beta correlation compared with individuals with lower SAD after presenting their good and bad characteristics. This effect reflects the immeasurability between the external part of the brain (cortex) and internal part (subcortex) during a post-event processing state. This is in line with the previous findings of cognitive-behavioral studies on individuals with SAD who participated in post-event rumination in the context of social events (Clark and McManus, 2002; Brozovich and Heimberg, 2008). According to the study by Poppelaars et al. (2018), frontal delta-beta is a stress-regulating indicator for females with test scores indicating high or low social anxiety. The results prove that delta-beta coupling distinguishes between HSA and LSA in the expectation phase of social performance tasks. LSA participants exhibit more significant differences under all conditions (resting, anticipation, and recovery), suggesting that frontal delta-beta is sensitive to trait anxiety and reflects an adaptive stress neural regulation mechanism (Poppelaars et al., 2018). Therefore, it seems worthwhile to increase the number of studies on the recovery state in social performance paradigms and future research should verify whether the delta-beta correlation during recovery can serve as a hypothetical EEG measurement of SAD. The aforementioned studies provide insight into the possibility of a neuronal delta-beta spectral correlate as quantification of the electrophysiological activity of neurons in post-event processing, and they provide evidence that the coherence between delta-beta sub-bands in SAD manifests is more strongly than any other frequency domain biomarker. The SW-FW coupling in anticipation and post-event states appears to be a more suitable candidate for electrical activity measurement in individuals with SAD. Empirically, the SW-FW coupling is thought to represent dynamic communication between the cortical (beta) and subcortical (delta) areas associated with anxiety (Knyazev et al., 2006; Knyazev, 2012). The increased SW-FW coupling in individuals with SAD is similar to conclusions of some fMRI studies, which found that the cortical and subcortical regions are more identical in individuals with GAD and even more so in individuals with SAD (Miskovic and Schmidt, 2012). The imbalance between the cortical layer and the subcortical layer is also consistent with the post-event procession bias found in psychotherapy studies (Kashdan and Roberts, 2007). Another significant finding is that the neural aggrandizement of the limbic (subcortical) and paralimbic (cortical) layers appear to be related to the functional mechanism (including more attentiveness toward the affective processing) of the social threats (Miskovic and Schmidt, 2012).

### Frontal Alpha EEG Asymmetry in Social Anxiety

The theory of hemispheric asymmetry and emotion is an influential theory that indicates the differences among individuals with different characteristics. The neural basis of emotions can be investigated via asymmetric patterns of EEG frontal alpha asymmetry power (Davidson, 1992). In particular, comparatively larger EEG left frontal power is associated with approach behaviors, while larger right EEG frontal power is associated with withdrawal behaviors (Meiers et al., 2020). Nonetheless, it should be noted that there is no straightforward consistency between withdrawal/approach effects and assertive/inhibition behaviors. For instance, anger is an assertive sensation associated with approach behaviors and is also found to be associated with higher left frontal cortical activity (Harrewijn et al., 2019). The frontal brain alpha asymmetry is usually expressed via the measurement of EEG alpha power over left frontal electrodes subtracted from the homologous electrodes of the right hemisphere (Davidson, 1998). Consequently, alpha power is inversely correlated to brain activity, with negative asymmetry power scores reflecting a higher relative right (i.e., reduced right frontal alpha power) and positive values reflect a stronger relative left frontal activation (i.e., reduced left frontal alpha power) (Zhang et al., 2020). Frontal EEG brain alpha asymmetry has been extensively investigated to examine the approached behaviors and emotional processing system in the brain (Harmon-Jones et al., 2010). In addition, frontal alpha asymmetry (FAA) has been proven to be involved in behavioral avoidance and inhibition (Allen et al., 2004). In contrast, many studies have confirmed that the relationship between FFA and behavior inhibition (social anxiety) is complex and uncorrelated (Coan and Allen, 2004). Thus, in Table 2 we have listed relevant EEG studies on frontal EEG alpha asymmetry.

**TABLE 2:** Summary of works about FAA related to SAD.

Frontal alpha asymmetry	Study	Participants	Task	EEG main findings
Resting state (RS)	Coles et al., 2002 Davidson et al., 2000 Miskovic et al., 2010 Harrewijn et al., 2019 Cohen and Gulbinaite, 2014 Moscovitch et al., 2011 Beaton et al., 2008 Schmidt, 2015 Roh et al., 2020	SAD people Pre to post CBT HSA vs. healthy Shy and SAD HSA vs. LSA HSA vs. LSA HSA vs. LSA HSA vs. LSA HSA vs. LSA MDD and HC	RS RS RS RS RS RS RS RS RS	Greater left frontal activity after CBT No significant differences between patients with SAD and control Increased right frontal power Decreased left frontal power Increase in left FAA after many psychological therapy. Greater right frontal lobe activity in adults with high scores of shyness. Greater relative right frontal EEG activity Greater relative right frontal EEG activity higher left frontal FAA in MDD more than HC
Anticipation	MacNamara et al., 2019 Coan and Allen, 2003 Davidson et al., 2000 Harrewijn et al., 2016 Schmidt, 2015 Cole et al., 2012 Wang et al., 2019	Shy and SAD subjects HSA vs. LSA HSA vs. LSA HSA vs. LSA HSA vs. LSA HSA vs. LSA SA vs. MDD	Anticipation Anticipation Anticipation Anticipation Anticipation Social task Treatment task	No significant difference Greater left frontal power activation in HAS Greater FAA scores in HAS No significant difference No significant difference FAA is more pronounced in LSA No significant difference
Mutual: resting anticipation recovery	Furmark, 2002 Miskovic et al., 2011a Harrewijn et al., 2016 Miskovic et al., 2011b Davidson et al., 2000 Coan and Allen, 2003 Schmidt, 2015 Beaton et al., 2008 Wang et al., 2019	Patients with SAD vs. control HSA vs. LSA HSA vs. LSA High vs. low socially withdrawn subjects HSA vs. LSA HSA vs. LSA HSA vs. LSA HSA vs. LSA SA vs. MDD	RS Anticipation Recovery RS Anticipation RS Anticipation Recovery RS Anticipation time Anticipation (Anticipation, Recovery) Recovery Anticipation Neurofeedback	No difference Greater in right frontal activity from baseline to anticipatory No significant difference No difference No significant difference. No significant difference No significant difference No significant difference No difference. Greater right prefrontal power in anticipatory No significant difference Decreased left frontal brain power compared to other states No significant difference Greater right frontal activity No significant difference

#### Frontal Alpha Asymmetry in the Resting State

FAA has been studied in the resting state, with participants asked to sit and blink or close their eyes for a certain period. Literature on the asymmetry of FAA in the resting baseline state in SAD appear to vary and are inconsistent (Roh et al., 2020). For instance, in one study individuals with SAD show an increase in left FAA after many psychological therapy sessions (Cohen and Gulbinaite, 2014). However, the study did not compare its results against control subjects. FAA in the resting state has also been studied in the context of symptoms associated with social anxiety, such as shyness, with higher right frontal lobe activity observed in adults with high scores on shyness than in those with low scores (Moscovitch et al., 2011). Conversely, other studies found no significant difference between individuals with SAD and normal HCs as well as between patients with severe and medium SAD in resting-state FAA (Davidson et al., 2000). It was also found that the drawn association between shyness and right frontal EEG alpha asymmetry in resting-state occurs only after controlling synchronous and concurrent depressive moods. After controlling the concomitant depression, individuals with high self-reported shyness and high socially withdrawn individuals have exhibit greater relative right frontal EEG activity at rest than slightly socially withdrawn individuals (Beaton et al., 2008; Schmidt, 2015; Harrewijn et al., 2016). In a very recent study, it was found that the FAA moderates the relationship between behavioral inhibition and the social effect of event-related negativity. FAA is not associated with behavioral inhibition or anxiety and it does not alleviate the relationship between early behavioral inhibition and subsequent anxiety symptoms (Cole et al., 2012). Participants with HSA and LSA differ significantly in FFA in resting-state. Participants with HAS also shows differences in the FFA in all anxious states, which means that FAA can be used as an indicator of SAD. A previous study (Harrewijn et al., 2019) showed greater left EEG frontal brain activation, which is thought to be correlated with approach behavioral but not with inhibition behaviors scores. Reduced left frontal activity is thought to reflect emotion regulation difficulties as reported in (Zhang et al., 2020).

#### Frontal Alpha Asymmetry in the Anticipation State

Cognitive models highlight the significance of emotional processing when individuals with SAD are anticipating being exposed to horrific social situations. Particularly, the anticipation time in SAD is investigated by giving an improvised self-presentation task in which the participant is requested to prepare a presentation about a general topic or private attributes. Individuals with SAD usually expect to engage in social interactions, which results in the production of more negative outcomes and more automatic negative emotional cycles in their social effectiveness. People with social anxiety are concerned that they will behave in an inappropriate manner, as this may lead to a negative evaluation by those around them (Hofmann, 2000; Clark and McManus, 2002; Hirsch et al., 2003). Scientifically it has always been posited that the greater left frontal power asymmetry is a constant biomarker of depression and anxiety and distinguishes between individuals with SAD and HC during any influential triggers (Coan and Allen, 2003). In a recent study (Coan and Allen, 2003), FFA was assessed by studying the neuronal differences between high and low SAD when participants engaged in established episode recalling tasks. The findings indicate that low individuals with SAD (trait anxiety) exhibit significantly higher right frontal EEG brain hemispheric activation than individuals with high or average SAD. Most studies seem to agree that FAA is associated with SAD during the time prior to involvement in social interaction (Davidson et al., 2000; Beaton et al., 2008). For instance, in a previous study (Davidson et al., 2000), the prefrontal FAA individuals with SAD was evaluated while they anticipated speaking on an unknown topic. SAD patients exhibited a higher right frontal activity during task preparation than in resting state. Similarly, participants with HSA exhibited elevated right frontal and lateral cortex power during the anticipation of a self-presentation while watching videotapes of the peers speaking in an anxious manner (Beaton et al., 2008). Previous investigations have shown no significant difference in the effect of SAD between people with severe anxiety and mild anxiety while they are expecting to give a speech (Schmidt, 2015; Harrewijn et al., 2016). Although (Schmidt, 2015; Poole and Schmidt, 2019) reported no significant differences between HSA and LSA, shyness was associated with an increase in right prefrontal activity (Wang et al., 2019), exclusively after managing the factors of mental stress. The dissimilarity in these neurophysiological EEG results can be explained by various interpretations. First, the effects of SAD can only be quantified at extreme grades of social anxiety elicited by stressors (verbal aggression). The impact of an extreme social stressor is more higher for severe SAD than mild SAD (Davidson et al., 2000). Nonetheless, 14 participants with SAD constitute a statistically small population to generalizing the results (Davidson et al., 2000); therefore, these results need to be interpreted with caution. Furthermore, only individuals with inhibited HSA show an increase in right prefrontal EEG alpha power. The group effect in the normal samples is found to be more pronounced when HSA participants are exposed to a socially frustrating tasks (watching the video of a peer) which may be expected in an upcoming event (Cole et al., 2012). The social tasks without stress-provoking circumstances may not cause an activated rise in FAA, as reported in a previous study (Harrewijn et al., 2016). Secondly, if the HC group does not show anxiety while performing a task, the effects of SAD may be significantly measurable. For instance, HC participants in a previous study (Davidson et al., 2000) did not exhibit an increase in subjective anxiety traits during anticipation, while in another study (Harrewijn et al., 2016). LSA participants recorded an increase in trait phobia scores. An increase in trait phobia scores among HCs may impede the identification of significant differences in FAA. Thirdly, some researchers (Beaton et al., 2008) focus on the distinction between two states (anticipation and resting states), while most studies focus only the anticipation state (Beaton et al., 2008; Schmidt, 2015). However, no significant effect of social anxiety has been reported between anticipation and resting-state data (Harrewijn et al., 2016). Fourth, the effect of SAD on FAA during the anticipatory time might be associated with the difference in anticipation time. In particular, some researchers used 3 min of anticipatory time and 2 min for planning sessions (Davidson et al., 2000), which may increase the social anxiety again at this stage. No studies have found frontal asymmetry effect (Harrewijn et al., 2016; Wang et al., 2019), even after using a comparatively long anticipation period (6 min). This is in contrast with the previously reported findings on asymmetrical effects (Schmidt, 2015). Generally, the ineffectiveness of tasks using prolonged anticipatory phase may be due to familiarization effects. Essentially, if the anticipatory time is long, individuals with SAD may get used to it and eventually show less positive activity. In future investigations, the potential familiarization impact on the tasks should be considered while comparing the FAA over different points during the anticipatory period or other states.

#### Frontal Alpha Asymmetry in the Recovery State

For individuals with SAD, recovering from social stress situations, such as delivering a public speech, may lead to increased anxiety induction in post-processing. As stated by several investigators (Clark and McManus, 2002; Brozovich and Heimberg, 2008), emotional post-event processing of SAD is marked by the reflection of continuous thoughtfulness (for example, unfavorable concerns about previous social interactions). Enhancing the painful memory retrieval and attention to negative presumptions are believed to retain symptoms of SAD (Morrison et al., 2019). Persistent rumination after a mental task (social stressor) can be investigated through FAA. So far, two studies have performed FAA measurement in the recovery state from self-presentation between HSA and LSA participants. Neuronal investigation (Davidson et al., 2000) has been unsuccessful for revealing the relationship between SAD and HCs individuals and FAA. The relationship between FAA and SAD was investigated in individuals with HSA and LSA (Harrewijn et al., 2016). Despite significant research deficiency, previous studies have suggested that FAA is not a candidate biomarker for emotional processing in participants with SAD in the recovery state because FAA cannot explain the emotional regulation in SAD. FAA during rest and post-event processing was not associated with SAD. However, the FAA during the anticipatory time seems to be a potential neuro-electrophysiological candidate biomarker of SAD, but only when anxiety is severe. Nevertheless, recent studies have found a decreased in depressive and anxiety traits after applying neurofeedback, but stable FAA over all the time (Wang et al., 2019). This may indicate that FAA is not a possible trait mark of SAD but may be related to SAD in the presence of some definite extreme stressors. A previous study (Thibodeau et al., 2006) in FAA-related literature, reported inconsistent findings, which indicate FFA may be a potential biomarker for other mental disorders such as major depressive disorder (MDD) and GAD. More studies need to focus on the coexistence of SAD and other mental disorders.

### ERPs Related to Information Processing in Social Anxiety

**FIGURE 3:**
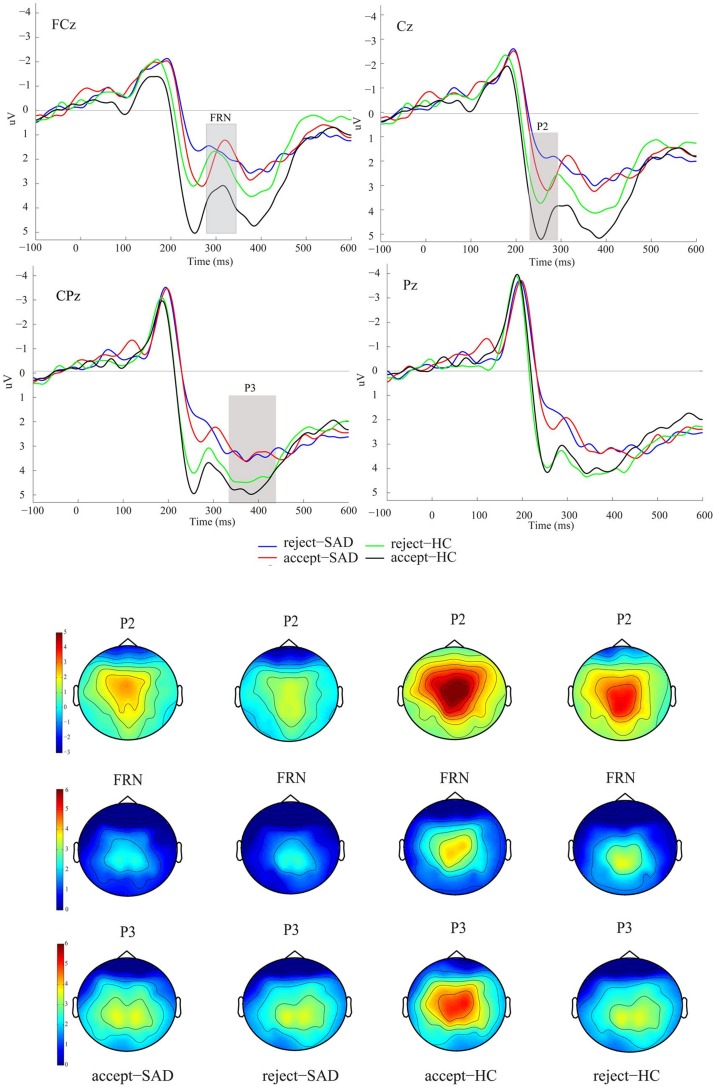
Grand averaged event-related potentials (ERPs) and topographic maps of the two feedback types for SAD and HC groups over midline electrodes (FCz, Cz, CPz, and Pz). [Four conditions: reject-SAD (blue), accept-SAD (red), reject-HC (green), and accept-HC (black)]. Reprinted from Frontiers in Psychology 6(204), Cao, Jianqin, Ruolei Gu, Xuejing Bi, Xiangru Zhu, and Haiyan Wu., Unexpected acceptance? Patients with social anxiety disorder manifest their social expectancy in ERPs during social feedback processing, 16; 6:1745, Copyright (2015), with permission from Frontier.

Event-related potential (ERPs) are scalp-recorded voltage fluctuations that measure brain response to a time-locked event (Herrmann and Knight, 2001). ERP reflects thousands of simultaneously ongoing brain processes that assess perceptual processing, attentional selection, cognition, and sensory-motor coupling. Generally, ERP studies are implemented in SAD research to describe electrocortical measurements associated with the stimulation processes involved in cognitive behavior and visual processing (Klawohn et al., 2020). ERP represents a change in electrocortical activities that have a time-locked for a specific stimulus and provide fine-grained description about the temporal structure of neural activation patterns (Koivisto and Revonsuo, 2010). ERP has the capability to allocate an objective insight to the immediate and later stages of neuronal stimulus processing (Herrmann and Knight, 2001). Figure 3, shows a typical ERP signal with the most common components in a study that employed self-relevant positive or negative social feedback (Cao et al., 2015). The ERPs triggered at 100 milliseconds after the stimulus are probably regulated by the physical factors of the stimulant rather than perceptual factors (Herrmann and Knight, 2001; Sa, 2005). However, highly prominent stimulants or alterations in the presentation sequence of the stimulus might affect these early ERP components (Meynadasy et al., 2019; de Bruijn et al., 2020), reflecting an influence on perception and attention (Eimer and Driver, 2001). SAD has shown a magnificent sensitivity to the amplitude of P1, as reported in certain studies (Hagemann et al., 2016; Fang et al., 2019; Torrence et al., 2019).

N1 (called N1) is the first negative-going component that is basically believed to capture premature perceptual coding and facial identification. N1 usually peaks at 130–200 milliseconds after the inception of the external stimulation triggers, primarily at the occipitotemporal lobe (Sa, 2005). Considerable research has been devoted to study the N1 component. These studies have emphasized that the N1 amplitude is correlated to visual stimulation and emotional expressions, while others have not shown this sensitivity to emotions (Kolassa and Miltner, 2006). Negative deflection ERP findings reveal that the first phases of visual processing may be affected by emotional mood expressions when the subject identifies different facial expressions. N1 does not appear to be regulated by SAD in reaction to a particular task. Participants with SAD and HCs do not show any significant difference in N1 amplitude as a neural reaction to emotional tasks (Kolassa and Miltner, 2006; Kolassa et al., 2009). In response to a visual task on emotional faces, N1 amplitudes showed no difference between HSA and LSA groups (Peschard et al., 2013; Hagemann et al., 2016). When classifying furious portraits in an emotional visual task, only one study showed that the N1 amplitude at the right parietal lobe increased in the SAD group and not in the HC group, which exhibited a smaller amplitude at the same locations (Peschard et al., 2013). It was concluded that the differences between HSA and HCs individuals were primarily observed in the early P1 and N1 components (Staugaard, 2010). In contrast with earlier studies, it was stated that SAD is correlated with higher P1 amplitude while the N1 is maintained at the same value (Hagemann et al., 2016). Recent studies on N1 amplitude show no difference between SAD and HCs individual in response to the facial expressions of happiness and fearfulness (Torrence et al., 2019).

P2 also called P200, is the second negative deflection that peaks at 150–250 milliseconds after the onset of anterior scalp stimulation (Schmidt, 2015), and is thought to reflect the post-synaptic activity of a neural process in the brain. The amplitude of P2 may be modulated by different aspects of visual stimuli, like perceptual processing and attention. In general the P2 amplitude is higher in response to a congruent target than in response to incongruent targets or uniform stimuli and the P2 component generally increases with unusual target stimuli (Kolassa et al., 2009; MacNamara et al., 2013). Usually, the P2 amplitude is higher as a reaction to happy and fearful stimuli than in response to neutral stimuli (MacNamara et al., 2013). The P2 amplitude increases in response to emotional faces, which can facilitate understanding of how prior information shapes future response, and it’s emotional importance in the cortex (Normann et al., 2007; MacNamara et al., 2013). When individuals with SAD were requested to concentrate their cognitive attentiveness on facial portraits, the P2 component appears to be sensitive to visual cognitive processing but not social anxiety. There is no significant difference in the P2 voltage value deflection between SAD and HC groups (Kolassa and Miltner, 2006). In addition, HSA and LSA individuals showed no difference in the P2 amplitude in a Stroop task (Peschard et al., 2013). People with HSA have not shown any attentional bias (Kolassa and Miltner, 2006). There was no difference in the P2 amplitude between the SAD and HC groups while processing a facial task (Hagemann et al., 2016). Furthermore, the second positive deflection component (P2) did not differ between the SAD and HC groups after a cognitive task (Kolassa et al., 2009), and HSA and LSA (Peschard et al., 2013). In this study, we further highlight the differences in these components and the underlying processing tasks as shown in Table 3 and investigate the effect of these attentional and cognitive tasks in individuals with SAD.

**TABLE 3:** Summary of works about ERPs related to SAD.

ERP related to SAD	Study	Participants	Applied Task	EEG main findings
P1	Luck, 2012 Peelen et al., 2007 Kolassa et al., 2009 Kolassa et al., 2007 Torrence et al., 2019 Hagemann et al., 2016 de Bruijn et al., 2020 Meynadasy et al., 2019 Fang et al., 2019	Control subjects Control subjects HSA vs. LSA HSA vs. LSA HSA vs. LSA HSA vs. LSA HSA vs. LSA HSA vs. LSA Maltreatment vs. non-maltreatment	Attention task Attention task Emotional faces Emotional expressions Facial stimuli Oddball paradigm (Masked faces) Flanker task Social threat task Facial stimulus	P1 amplitude is greater on the lateral occipital area. Activation of the lateral occipital cortex SAD individuals show an increment in P_1_ value Increased P1 amplitude in SAD Increased P1 amplitude in SAD P1 was higher in the masked conditions P1 amplitude was higher in the harm conditions P1 amplitude was higher than LSA P1 amplitude is greater in the non-treatment group
N1	Kolassa et al., 2009 Kolassa and Miltner, 2006 Peschard et al., 2013 Torrence et al., 2019 Hagemann et al., 2016 Fang et al., 2019	HSA vs. LSA HSA vs. LSA HSA vs. LSA HSA vs. LSA HSA vs. LSA Maltreatment vs. non-maltreatment	Identifying Stroop task Modified Stroop task Visual task of emotional faces Facial stimuli Oddball paradigm Facial stimulus	SAD people and HC did not show any significant differences in N170 amplitude N170 amplitudes have no difference in HSA and LSA. N1 has no differences No changes in N170 amplitude. More enhanced N170 amplitudes N1 amplitude is greater in the treatment group
P2	Peschard et al., 2013 Luck, 2012 Normann et al., 2007 Kolassa et al., 2009 Hagemann et al., 2016 MacNamara et al., 2019	High vs. low HC individuals HC participants HSA vs. LSA SAD vs. control HSA vs. LSA	Emotional faces Stroop task Emotional faces Emotional faces Modified Stroop task Face learning task Emotional faces	LSAs have no difference in P2 from LSA P2 increases with unusual target stimuli P2 voltage value increased as reaction to schematic expression HSAs have no difference in P2 from LSA HSAs have no difference in P2 from LSA SAD showed increased late P2 amplitude to negative stimuli
VEPs	Di Russo et al., 2002 McNair et al., 2006 Fotiou et al., 2003 Azoulay et al., 2020 de Bruijn et al., 2020 MacNamara et al., 2019	HC participants HC participants MDD HSA vs. LSA HSA vs. LSA HSA vs. LSA	Checkerboard reversal stimulus Checkerboard stimulus Checkerboard stimulus Facial stimulus Flanker task Emotional faces	The amplitude of P1 and N1 increased after onset Enhancement of the N1 of the VEPs A positive correlates between the latency of N80 and P100 Latency is negatively related to SAD severity SA is sensitive loud noise more aversive than the soft noise SAD showed increased late VEPs amplitude to negative stimuli

VEPs constitute an ideal tool for recording the strength of visual processing of the occipital electrodes and to quantify cortical potential correlates of attention distribution during EEG recording from the scalp overlying visual cortex simultaneously. In a previous study (Normann et al., 2007), VEP was used as an attainable paradigm for studying cortical potential activations in the brain. VEPs are simultaneously stimulating the mean EEG signals that primarily reflect neuronal excitatory and inhibitory synapses and action potentials from various sensory information sites. The integration of VEP data and fMRI modality leads to an electrocortical activity source by tracking the activation of the visual cortex in the first VEP components (Di Russo et al., 2002). The results reported show that long-term visual stimulation generates a plastic alteration in the cortical response, and the amplitude of P1 and N1 increase significantly after the onset of stimuli induction. In some experimental studies (Di Russo et al., 2002; McNair et al., 2006; Normann et al., 2007), a 9 Hz checkerboard reversal stimulus used for 2 min resulted in an enhancement of the N1 amplitude of the VEP.

Reportedly, the latency of N1 and P1 in atypical patients is significantly shortened, while in patients with depression is longer (Fotiou et al., 2003). A positive relationship between the latency of N1 and P1 was proven, and the depression index was negatively correlated with the occurrence of feared social interaction. A greater negative relationship was reported between pattern-reversed latency and feared social interaction scores. Generally, individuals with SAD have a capability for detecting the faster offset visual stimulus compared to LSA as reported recently (Azoulay et al., 2020), which means detection latency is negatively related to SAD severity.

Finally, it may be concluded that several types of studies on SAD have proven that SAD is more strongly associated with an increment in P1 and late positive latency amplitude and constancy of N1 amplitude (MacNamara et al., 2019). It must be emphasized that most of the previous experiments comprised a small-sized population (10–22 for SAD), which is insufficient for generalizing the final neuronal outcomes. The association between SAD and ERP findings have been explored more deeply in a previous review (Harrewijn et al., 2017). In addition to ERP studies, recently, SAD has been studied via changes in connectivity diagrams between various sites in the brain. In the subsequent section, we outline the various types of connectivity and summarize up-to-date findings on SAD based on these.

### Brain Connectivity

Recently, there has been growing interest among researchers in studying both normal and pathological brain functions not only through variations in activation between brain areas, but also via interactions among the neural assemblies dispersed over different brain regions (Beaty et al., 2019). This network of interactions between various regions of the brain is called brain connectivity (Busby et al., 2019). Brain connectivity can be explained in terms of structural, functional, and effective connectivity. Fiber pathways physically extend from one brain region to another, representing structural connectivity (Sokolov et al., 2019). These fiber tracts can be best observed using MRI and diffusion tensor imaging (DTI) technique (Richards et al., 2015). Apart from being structurally connected, brain regions may become functionally connected, i.e., the neuronal activities among different brain regions become statistically dependent while performing a function. This type of statistical dependence is commonly referred to as functional connectivity (FC) (Greicius et al., 2009). To avoid the fundamental drawbacks of FC, in terms of its bidirectional nature and susceptibility to connection with a third party, a relatively new concept called effective connectivity (EC), was proposed (Valdes-Sosa et al., 2011). EC reflects the causal interaction between the driver (initiating external force) and the response (the driven system); it has a more direct influence on one neural system of a brain region than over others and defines dynamic directional interaction among them (Valdes-Sosa et al., 2011). This influence can be directly estimated through signals and called data-driven EC or is named based on a model that specifies causal links among different brain regions, termed model-driven EC (Bakhshayesh et al., 2019).

In the two subsequent subsections, recent studies and findings on SAD using FC and EC of the brain are outlined as shown in Table 4.

**TABLE 4:** Summary of works about brain connectivity related to SAD.

Connectivity	Study	Participants	Main findings
Functional connectivity	Xing et al., 2017 Yuan et al., 2016 Bhaumik et al., 2017 Imperatori et al., 2019 Prater et al., 2013 Qiu et al., 2011 Hahn et al., 2011 Etkin and Wager, 2007 Phan et al., 2006 Kawaguchi et al., 2016 Klumpp et al., 2012	gSAD and HC gSAD and HC HAS vs. HC HAS vs. HC HAS vs. HC HAS vs. HC HAS vs. HC HAS vs. HC HAS vs. HC HAS vs. HC HAS vs. HC	The WPLI findings show an increase in the mid-frontal coherence in the theta rhythm, showing that the gSAD has higher connectivity at resting compared to the HC group. GSAD patients display high decreased connection in the amygdala compared with baseline after application of CBT. Unanimity links used to differentiate SAD subjects are broadly located within or through the DMN Lower beta FC is shown in the right and the right amygdala and anterior cortex HSA displayed decreased FC between the amygdala and cortex compared to HC while watching fearful faces. Relative to HC, SAD individuals show less connection in gyrus and the left cortical sites within the DMN Greater functional amygdala activation Greater functional amygdala activation in response to social stimuli Greater functional amygdala activation response to harsh faces Bilateral insular volume was reduced in patients with SAD Insula-prefrontal connectivity has shown a higher hyperactivity to threat stimulus in SAD individuals compared to HC
Effective connectivity	Liao et al., 2010 Sladky et al., 2013 Freitas-Ferrari et al., 2010 Engel et al., 2009 Shahabi and Moghimi, 2016 Bae et al., 2017	HAS vs. HC HAS vs. HC HAS vs. HC HAS vs. HC HAS vs. HC Alcoholics vs. normal subjects	In SAD, it was found that the effect of gyrus to amygdala is decreased, while the causal effect in the amygdala and the occipital cortex was greater than HC. SAD people, obvious correlate from the frontal cortex to the amygdala was observed, suggesting the presence of excitatory connectivity. An abnormal amygdala in SAD patients. Also an abnormal amygdala in SAD patients The perceived valence was positively correlated with the frontal inter-hemispheric flow but negatively correlated with the parietal bilateral connectivity Findings reveal a distinguished impacts in addictive alcohol compared to HC

#### Functional Connectivity

Functional connectivity in neuroscience represents simultaneous activities performed within different anatomical units of the brain. Two regions are considered functionally connected if their activities within a nervous system are highly correlated over time. FC is basically a statistical connotation, and it captures variations in statistical dependence for distributed remote neuronal units. Statistical dependence is excessively applied to delineate regions of the brain that alter their level of activation in reaction to any neural stimuli or cognitive task and can be determined by quantifying correlation matrices, coherence or phase-locking. Among the modalities used for FC estimation, EEG is preferable because it has a higher temporal resolution than other neuroimaging techniques, such as fMRI. FC estimation is mostly dependent on the use of the resting-state fMRI data of different brain sites. The level of FC is usually quantified between all neural activity units in the brain, irrespective of whether they are linked by linear connections. Unlike structural connectivity, FC is highly influenced by time: cortical oscillation patterns statistically fluctuate in several specific period ranges at the same time (10–100 ms). It is worth noting that FC does not explicitly designate a particular directional effect or basic physical pattern. Contemporary studies have found that interregional connections between different units of circuits in each of the visual, motor, language, and working memory systems can be revealed in the resting-state default network mode (DMN). Because of the crucial emotional processing functions of the limbic system in the brain, many researches consider the amygdala and insula as prime regions of interest for analysis of the FC of individuals with social anxiety. In a recent study on SAD, using the FC of the resting-state network, the EEG data of 32 patients with generalized social anxiety disorder (gSAD) and 32 statistically HCs were recorded (Xing et al., 2017). Application of weighted phase lag index (WPLI) to EEG electrodes showed an increase in the enhanced fluctuation in oscillatory theta- rhythm coherence in the mid-frontal regions, proving that the gSAD has a higher connectivity in the resting-state in SAD patients than in HCs. Fifteen individuals with gSAD showed a considerably lower FC of the left limbic unit (amygdala) relative to baseline after application of CBT (Zhou et al., 2017). The multivariate pattern algorithms in fMRI classification analysis were applied to 20 SAD and 20 HCs individuals, and showed that the unanimity of the links used to differentiate socially anxious subjects are broadly located within or across the DMN, sensory-motor neurons, visual networking, and salience networks (Bhaumik et al., 2017). As expected, SAD patients recorded an excessive activation of the amygdala in emotional paradigms and reduced functional coupling of the left amygdala (Hahn et al., 2011). Similarly, patients with SAD have consistently exhibited greater amygdala activation in response to potential social stimuli, negative emotions (Etkin and Wager, 2007), and harsh facial perception (Birbaumer et al., 1998; Phan et al., 2006). A decrease in beta connectivity was also observed between the left and right anterior cingulate cortex (Imperatori et al., 2019). However, because of the small sample size of the previous study, we believe it is impossible to reach a definitive conclusion about the finalized FC findings on the SAD and HC groups. In addition, this study did not determine the alterations in the state of anxiety, which makes this interpretation specific to trait anxiety. Furthermore, the insular cortex (insula) has also been experimentally demonstrated to be hyperactive in individuals with SAD, and the activation strength is believed to be related to the level of anxiety. Insular cortex activity has been shown to be decreased after applying appropriate antianxiety treatment. For instance, two MRI studies have been conducted to examine and compare the insula activation in individuals with SAD against HC individuals, and the findings established a statistically significant difference between the insular volume for the SAD and HC groups. Bilateral insular volume was reduced in SAD patients, suggesting that abnormal neuroanatomical networks exist in individuals with SAD (Syal et al., 2012; Kawaguchi et al., 2016). Another FC study has demonstrated that the insula is a communication link between internal and external information for creating an awareness of an internal body sensation. Aberrant insula-prefrontal connectivity has shown higher hyperactivity to threat stimulus in individuals with SAD compared to HCs (Klumpp et al., 2012). Recently, functional MRI studies have reported that the individuals with SAD group exhibit greater activation in the bilateral posterior insula during a social performing task compared to HCs, which may reflect premature markers in self-perceptual SAD (Wang et al., 2019; Yuan et al., 2020). Functional neuroimaging experiments on SAD focus on regional neural activity in response to anxiety, provocation, or emotional facial cues.

Though, fMRI and EEG techniques have emerged as powerful tools for mapping large-scale networks in the human brain, they have some important limitations. These limitations include the determination of the networking nodes, connection analysis for every individual node, voxel-level global brain estimation, node groups, and FC variations. FC does not specifically elucidate the directional influence of the neuronal signal in the brain. Critically to examine the variability between groups, FMRI is sensitive to head motion and variations in mental states during scans.

#### Effective Connectivity

Effective connectivity is recognized as a combination of physical connections and FC because EC can effectively identify the network of causal influences of one neural unit over others within the brain. Basically, dynamic influences are deduced by the methodical disturbance of the model because reasons must exceed results in timing via mathematical time-domain analysis. Several methods used to extract EC parameters require a specific system that implicates physical patterns. Various effective features of EEG (high temporal resolution, inexpensive device, and portability) make it an appropriate modality for studying the neural connections of cognitive function. Previously, EEG power spectra have been studied to clarify changes in different frequency bands in emotional processing. Functional brain connections might be quantified by a variety of techniques, e.g., calculating mutual information, cross-correlation, interrelations between EEG signals, or coherence in the time and frequency domain. Calculating EC is more challenging than determining functional brain connectivity. As mentioned earlier, FC quantifies statistically dependent patterns, while EC determines a network of the directed influence of one neuronal unit over others. Several mathematical models for evaluating EC are continuously being investigated and developed. A model called covariance structural equation modeling allocates EC intensity to structural paths, which is perfectly similar to the covariance found in any specific experiment (Friston et al., 2003). The generalization of this process is known as dynamic causal modeling (DCM) (Tononi and Sporns, 2003), which functions in a Bayesian structured sample to assess and conclude the causal effects in neural elements. Different methods are used to identify extensive brain interactions between various neural sites and their causal links, including the effective data-driven approach that uses a perturbation method to capture the extent to which two brain regions interact with each other (Liao et al., 2010). It is also possible to estimate effective connections using time series analysis. Some of these methods are based on the interpretation concept of the Granger causality (GC) (Ding et al., 2006; Seok and Cheong, 2019). Furthermore, transfer entropy causality measure has been used extensively (Schreiber, 2000), and aims to reveal the reciprocation of directional input data flowing in neural units by considering the effectiveness of the condition of one neural unit based on the state transference probability of another neural unit. EC causality has a high sensitivity to data reduction, sampling rate, windowing patterns, and state-space selection. An fMRI-based resting state study (Liao et al., 2010) was the first study to reveal and quantify an abnormal brain network via the assessment of EC in individuals with SAD. The effect of the ventral cerebral cortex on the amygdala was found to be reduced in the SAD group significantly more than in the HC group, while the causal effect between the limbic system (amygdalan components) and the visual cortex was increased using GC analysis. In a previous study (Sladky et al., 2013), SAD patients showed a positive correlation between the frontal cortex and the amygdala, which indicates the presence of excitatory connectivity. An abnormal amygdala is often found in patients with SAD (Engel et al., 2001; Freitas-Ferrari et al., 2010) based on EC. A resting-state FMRI data-based study (Bae et al., 2017) proposed a network attribute model based on GC for investigating the EC of patients with GAD and HCs. Consequently, it was reported that individuals with GAD have a reduced EC from the cortical regions to the amygdala (Qiao et al., 2017). In a previous study (Minkova et al., 2017), 15 patients with SAD and 15 HCs were recruited during a cognitive task with emotional scenes to estimate the causal EC of affective stimuli in the amygdala and prefrontal cortical neurons. The amygdala activation was found to be higher after a low cognitive load in individuals with SAD than in HCs. EC might be influenced by the cognitive load during cognitive task processing and result in a different EC in SAD participants from that HCs. Recently, in Dong et al. (2019), 35 GAD patients and 36 HCs underwent resting-state fMRI with GC analysis to compare and investigate whole brain connectivity and the amygdala via EC. The results demonstrate disrupted GC influences from the left to the right of the amygdala in individuals with GAD relative to HCs. This study (Makovac et al., 2016) shows significantly higher connectivity alteration across the entire in SAD patients than in HCs. Patients with SAD exhibited a comparatively lower neural connection between both sides of the amygdala and the right gyrus than HCs. In addition, the EC between the anterior insula and prefrontal cortex has been investigated to explore the activation differences in 20 SAD participants and 20 matched HCs. It was recently revealed that anxious individuals exhibited significantly decreased EC between the insula and prefrontal cortex (Kandilarova et al., 2018).

To date, among the different brain imaging techniques, the fMRI modality is more dominant and more extensively used to investigate EC in SAD than any other neuroimaging technique. EEG has several merits when exploring neural activity. First, EEG has an excellent temporal resolution (millisecond or lesser), which allows the system to detect and capture any sudden changes in electrical activity. Second, a set of EEG electrodes are attached to the scalp of the subject, making the EEG technique non-invasive because no interventional medical surgery is required. Third, EEG equipment is relatively inexpensive, easy to operate, and clinically available. Fourth, the EEG technique can quantify the EC between different regions of the brain without physiological information and clarify how activities in distinct brain areas affect each other.

## Conclusion and Recommendations

In summary, SAD is a prevalent severe intellectual abnormality. Unfortunately, the diagnosis of SAD is still date obstinately built on personal evaluation and traditional procedures. The effects of SAD on cognitive behavior and oscillatory dynamics in social interaction models are investigated in SAD patients and HCs. Power spectral density analysis of EEG signals and brain connectivity estimators are encouraging techniques for illustrating the neuronal correlations of SAD and may provide an expedient substitute to conventional brain biomarkers. Despite extensive studies on SAD, further research is needed to validate the current results and further evaluate the predictive social anxiety biomarkers. These neural biomarkers observed during traumatic situations are highly effective for the exploration of the genetic basis of social anxiety, improvement of psychological therapy, and early identification of individuals possibly at risk of SAD. However, the improved techniques for these purposes are ineffective because the lower prognostic precisions or final outcomes cannot be generalized because for a small sample size. Another challenge is the advocation of previous empirical facts and contextual mechanisms to be addressed if rectifying these cognitive behaviors can restrain the expansion of SAD in individuals with genetic susceptibility. The following recommendations highlight the significance of this study and its usefulness to future search in avoiding potential challenges.

1. The selection of a sample size is a vital determinant of experimental quality because it ascertains the significance of the datasets and minimizes typical errors. It is difficult to generalizing the findings of some studies and to draw a clear conclusion about the final conclusions because of the small sample size.
2. Several biomarkers discussed in this study, such as the delta-beta correlation, FAA, midline theta, and brain connectivity, can be studied in combinations to validate the main findings and to devise effective diagnostic methods.
3. Multimodal neuroimaging techniques are extensively applied in neuroscience to better capture and visualize neuronal network activation. For instance, a model that involves the simultaneous measurement of PET, CT, EEG, fMRI, and MEG, EEG-fNIR, or fMRI and MEG can be applied. EEG-fMRI integration will result in a greater level of temporal resolution and more accurate data on the dynamic processes of the brain.

## Author Contributions

NK and IF devised the project, the main conceptual ideas, and proof outline. EG supervised the psychological part of the experiment. AA-E suggested the experimental design and wrote the manuscript.

## Conflict of Interest

The authors declare that the research was conducted in the absence of any commercial or financial relationships that could be construed as a potential conflict of interest.

## References

[B1] Abbott M. J. Rapee R. M. (2004). Post-event rumination and negative self-appraisal in social phobia before and after treatment. *J. Abnorm. Psychol.* 113:136. 14992666 10.1037/0021-843X.113.1.136

[B2] Allen J. J. B. Coan J. A. Nazarian M. (2004). Issues and assumptions on the road from raw signals to metrics of frontal EEG asymmetry in emotion. *Biol. Psychol.* 67 183–218. 15130531 10.1016/j.biopsycho.2004.03.007

[B3] Alvares G. A. Quintana D. S. Kemp A. H. Van Zwieten A. Balleine B. W. Hickie I. B. (2013). Reduced heart rate variability in social anxiety disorder: associations with gender and symptom severity. *PLoS One* 8:e70468. 10.1371/journal.pone.0070468 23936207 PMC3728204

[B4] Arditte Hall K. A. Quinn M. E. Vanderlind W. M. Joormann J. (2019). Comparing cognitive styles in social anxiety and major depressive disorders: an examination of rumination, worry, and reappraisal. *Br. J. Clin. Psychol.* 58 231–244. 10.1111/bjc.12210 30484868 PMC6470033

[B5] Azoulay R. Berger U. Keshet H. Niedenthal P. M. Gilboa-Schechtman E. (2020). Social anxiety and the interpretation of morphed facial expressions following exclusion and inclusion. *J. Behav. Ther. Exp. Psychiatry* 66 101511. 10.1016/j.jbtep.2019.101511 31614264

[B6] Bae Y. Yoo B. W. Lee J. C. Kim H. C. (2017). Automated network analysis to measure brain effective connectivity estimated from EEG data of patients with alcoholism. *Physiol. Meas.* 38 759. 10.1088/1361-6579/aa6b4c 28448272

[B7] Bakhshayesh H. Fitzgibbon S. P. Janani A. S. Grummett T. S. Pope K. J. (2019). Detecting connectivity in EEG: a comparative study of data-driven effective connectivity measures. *Comput. Biol. Med.* 111 103329. 10.1016/j.compbiomed.2019.103329 31425938

[B8] Baptista C. A. Loureiro S. R. de Lima Osório F. Zuardi A. W. Magalhães P. V. Kapczinski F. (2012). Social phobia in Brazilian university students: prevalence, under-recognition and academic impairment in women. *J. Affect. Disord.* 136 857–861. 10.1016/j.jad.2011.09.022 22018945

[B9] Beaton E. A. Schmidt L. A. Ashbaugh A. R. Santesso D. L. Antony M. M. McCabe R. E. (2008). Resting and reactive frontal brain electrical activity (EEG) among a non-clinical sample of socially anxious adults: does concurrent depressive mood matter? *Neuropsychiatr. Dis. Treat.* 4 187–192. 18728822 10.2147/ndt.s1388 PMC2515916

[B10] Beaty R. E. Seli P. Schacter D. L. (2019). Network neuroscience of creative cognition: mapping cognitive mechanisms and individual differences in the creative brain. *Curr. Opin. Behav. Sci.* 27 22–30. 10.1016/j.cobeha.2018.08.013 30906824 PMC6428436

[B11] Bhaumik R. Jenkins L. M. Gowins J. R. Jacobs R. H. Barba A. Bhaumik D. K. (2017). Multivariate pattern analysis strategies in detection of remitted major depressive disorder using resting state functional connectivity. *Neuroimage* 16 390–398. 10.1016/j.nicl.2016.02.018 28861340 PMC5570580

[B12] Birbaumer N. Grodd W. Diedrich O. Klose U. Erb M. Lotze M. (1998). fMRI reveals amygdala activation to human faces in social phobics. *Neuroreport* 9 1223–1226. 9601698 10.1097/00001756-199804200-00048

[B13] Bishop S. J. (2007). Neurocognitive mechanisms of anxiety: an integrative account. *Trends Cogn. Sci.* 11 307–316. 17553730 10.1016/j.tics.2007.05.008

[B14] Blöte A. W. Miers A. C. den Bos E. Westenberg P. M. (2019). The role of performance quality in adolescents’ self-evaluation and rumination after a speech: is it contingent on social anxiety level? *Behav. Cogn. Psychother.* 47 148–163. 29769157 10.1017/S1352465818000310

[B15] Brooker R. J. Phelps R. A. Davidson R. J. Goldsmith H. H. (2016). Context differences in delta beta coupling are associated with neuroendocrine reactivity in infants. *Dev. Psychobiol.* 58 406–418. 10.1002/dev.21381 26566605 PMC4801734

[B16] Brown M. Stopa L. (2007). Does anticipation help or hinder performance in a subsequent speech? *Behav. Cogn. Psychother.* 35 133–147.

[B17] Brozovich F. Heimberg R. G. (2008). An analysis of post-event processing in social anxiety disorder. *Clin. Psychol. Rev.* 28 891–903. 10.1016/j.cpr.2008.01.002 18294745

[B18] Busby N. Halai A. D. Parker G. J. M. Coope D. J. Ralph M. A. L. (2019). Mapping whole brain connectivity changes: the potential impact of different surgical resection approaches for temporal lobe epilepsy. *Cortex* 113 1–14. 10.1016/j.cortex.2018.11.003 30557759

[B19] Cao J. Gu R. Bi X. Zhu X. Wu H. (2015). Unexpected acceptance? Patients with social anxiety disorder manifest their social expectancy in ERPs during social feedback processing. *Front. Psychol.* 6:1745. 10.3389/fpsyg.2015.01745 26635659 PMC4644791

[B20] Clark D. M. McManus F. (2002). Information processing in social phobia. *Biol. Psychiatry* 51 92–100. 11801234 10.1016/s0006-3223(01)01296-3

[B21] Clark D. M. Wells A. (1995). A cognitive model of social phobia. *Soc. Phobia* 41 22–23.

[B22] Coan J. A. Allen J. J. B. (2003). Frontal EEG asymmetry and the behavioral activation and inhibition systems. *Psychophysiology* 40 106–114. 12751808 10.1111/1469-8986.00011

[B23] Coan J. A. Allen J. J. B. (2004). Frontal EEG asymmetry as a moderator and mediator of emotion. *Biol. Psychol.* 67 7–50. 15130524 10.1016/j.biopsycho.2004.03.002

[B24] Cohen M. X. Gulbinaite R. (2014). Five methodological challenges in cognitive electrophysiology. *Neuroimage* 85 702–710. 10.1016/j.neuroimage.2013.08.010 23954489

[B25] Cole C. Zapp D. J. Katherine Nelson S. Pérez-Edgar K. (2012). Speech presentation cues moderate frontal EEG asymmetry in socially withdrawn young adults. *Brain Cogn.* 78 156–162. 10.1016/j.bandc.2011.10.013 22169714 PMC3268053

[B26] Coles M. E. Turk C. L. Heimberg R. G. (2002). The role of memory perspective in social phobia: immediate and delayed memories for role-played situations. *Behav. Cogn. Psychother.* 30 415–425.

[B27] Condren R. M. O’Neill A. Ryan M. C. M. Barrett P. Thakore J. H. (2002). HPA axis response to a psychological stressor in generalised social phobia. *Psychoneuroendocrinology* 27 693–703. 12084662 10.1016/s0306-4530(01)00070-1

[B28] Costello E. J. Mustillo S. Erkanli A. Keeler G. Angold A. (2003). Prevalence and development of psychiatric disorders in childhood and adolescence. *Arch. Gen. Psychiatry* 60 837–844. 12912767 10.1001/archpsyc.60.8.837

[B29] Davidson R. J. (1992). Anterior cerebral asymmetry and the nature of emotion. *Brain Cogn.* 20 125–151. 1389117 10.1016/0278-2626(92)90065-t

[B30] Davidson R. J. (1998). Affective style and affective disorders: perspectives from affective neuroscience. *Cogn. Emot.* 12 307–330.

[B31] Davidson R. J. Marshall J. R. Tomarken A. J. Henriques J. B. (2000). While a phobic waits: regional brain electrical and autonomic activity in social phobics during anticipation of public speaking. *Biol. Psychiatry* 47 85–95. 10.1016/S0006-3223(99)00222-X 10664824

[B32] de Bruijn E. R. A. Jansen M. Overgaauw S. (2020). Enhanced error-related brain activations for mistakes that harm others: ERP evidence from a novel social performance-monitoring paradigm. *Neuroimage* 204:116238. 10.1016/j.neuroimage.2019.116238 31585173

[B33] De Pascalis V. Vecchio A. Cirillo G. (2020). Resting anxiety increases EEG delta–beta correlation: relationships with the reinforcement sensitivity theory personality traits. *Pers. Ind. Diff.* 156:109796.

[B34] Di Russo F Martínez A. Sereno M. I. Pitzalis S. Hillyard S. A. (2002). Cortical sources of the early components of the visual evoked potential. *Hum. Brain Mapp.* 15 95–111. 11835601 10.1002/hbm.10010 PMC6871868

[B35] Ding M. Chen Y. Bressler S. L. (2006). “Granger causality: basic theory and application to neuroscience,” in *Handbook of Time Series Analysis: Recent Theoretical Developments and Applications*, eds Schelter B. Winterhalder M. Timmer J. (Hoboken, NJ: John Wiley and Sons, Inc).

[B36] Dong M. Xia L. Lu M. Li C. Xu K. Zhang L. (2019). A failed top-down control from the prefrontal cortex to the amygdala in generalized anxiety disorder: evidence from resting-state fMRI with Granger causality analysis. *Neurosci. Lett.* 707:134314. 10.1016/j.neulet.2019.134314 31163226

[B37] Edwards S. L. Rapee R. M. Franklin J. (2003). Postevent rumination and recall bias for a social performance event in high and low socially anxious individuals. *Cogn. Ther. Res.* 27 603–617.

[B38] Eimer M. Driver J. (2001). Crossmodal links in endogenous and exogenous spatial attention: evidence from event-related brain potential studies. *Neurosci. Biobehav. Rev.* 25 497–511. 11595270 10.1016/s0149-7634(01)00029-x

[B39] Engel K. Bandelow B. Gruber O. Wedekind D. (2009). Neuroimaging in anxiety disorders. *J. Neural Trans.* 116 703–716. 10.1007/s00702-008-0077-9 PMC2694920 18568288

[B40] Engel A. K. Fries P. Singer W. (2001). Dynamic predictions: oscillations and synchrony in top–down processing. *Nat. Rev. Neurosci.* 2:704. 11584308 10.1038/35094565

[B41] Etkin A. Wager T. D. (2007). Functional neuroimaging of anxiety: a meta-analysis of emotional processing in PTSD, social anxiety disorder, and specific phobia. *Am. J. Psychiatry* 164 1476–1488. 17898336 10.1176/appi.ajp.2007.07030504 PMC3318959

[B42] Fang J. Wang S. Liu J. Gong J. (2019). Early ERP components to emotional facial expressions in young adult victims of childhood maltreatment. *Psychiatry Res.* 275 120–128. 10.1016/j.psychres.2019.03.024 30901670

[B43] Field A. P. Psychol C. Morgan J. (2004). Post-event processing and the retrieval of autobiographical memories in socially anxious individuals. *J. Anxiety Disord.* 18 647–663. 15275944 10.1016/j.janxdis.2003.08.004

[B44] Foley P. Kirschbaum C. (2010). Human hypothalamus–pituitary–adrenal axis responses to acute psychosocial stress in laboratory settings. *Neurosci. Biobehav. Rev.* 35 91–96. 10.1016/j.neubiorev.2010.01.010 20109491

[B45] Ford T. Goodman R. Meltzer H. (2003). The British child and adolescent mental health survey 1999: the prevalence of DSM-IV disorders. *J. Am. Acad. Child Adolesc. Psychiatry* 42 1203–1211. 14560170 10.1097/00004583-200310000-00011

[B46] Fotiou F. Fountoulakis K. N. Iacovides A. Kaprinis G. (2003). Pattern-reversed visual evoked potentials in subtypes of major depression. *Psychiatry Res.* 118 259–271. 12834820 10.1016/s0165-1781(03)00097-0

[B47] Frandsen F. W. Simonsen S. Poulsen S. Sørensen P. Lau M. E. (2019). Social anxiety disorder and avoidant personality disorder from an interpersonal perspective. *Psychol. Psychother.* 93 88–104. 10.1111/papt.12214 30656823

[B48] Freitas-Ferrari M. C. Hallak J. E. C. Trzesniak C. Santos Filho A. Machado-de-Sousa J. P. Chagas M. H. N. (2010). Neuroimaging in social anxiety disorder: a systematic review of the literature. *Prog. Neuro Psychopharmacol. Biol. Psychiatry* 34 565–580. 10.1016/j.pnpbp.2010.02.028 20206659

[B49] Friston K. J. Harrison L. Penny W. (2003). Dynamic causal modelling. *Neuroimage* 19 1273–1302. 12948688 10.1016/s1053-8119(03)00202-7

[B50] Furmark T. (2002). Social phobia: overview of community surveys. *Acta Psychiatr. Scand.* 105 84–93. 11939957 10.1034/j.1600-0447.2002.1r103.x

[B51] Givon-Benjio N. Okon-Singer H. (2020). Biased estimations of interpersonal distance in non-clinical social anxiety. *J.Anxiety Disord.* 69 102171. 10.1016/j.janxdis.2019.102171 31865274

[B52] Grant B. F. Hasin D. S. Blanco C. Stinson F. S. Chou S. P. Goldstein R. B. (2005). The epidemiology of social anxiety disorder in the United States: results from the national epidemiologic survey on alcohol and related conditions. *J. Clin. Psychiatry* 66 1351–1361. 16420070 10.4088/jcp.v66n1102

[B53] Greicius M. D. Supekar K. Menon V. Dougherty R. F. (2009). Resting-state functional connectivity reflects structural connectivity in the default mode network. *Cereb. Cortex* 19 72–78. 18403396 10.1093/cercor/bhn059 PMC2605172

[B54] Hagemann J. Straube T. Schulz C. (2016). Too bad: bias for angry faces in social anxiety interferes with identity processing. *Neuropsychologia* 84 136–149. 10.1016/j.neuropsychologia.2016.02.005 26878979

[B55] Hahn A. Stein P. Windischberger C. Weissenbacher A. Spindelegger C. Moser E. (2011). Reduced resting-state functional connectivity between amygdala and orbitofrontal cortex in social anxiety disorder. *Neuroimage* 56 881–889. 10.1016/j.neuroimage.2011.02.064 21356318

[B56] Hansen R. A. Gaynes B. N. Gartlehner G. Moore C. G. Tiwari R. Lohr K. N. (2008). Efficacy and tolerability of second-generation antidepressants in social anxiety disorder. *Int. Clin. Psychopharmacol.* 23:170. 10.1097/YIC.0b013e3282f4224a 18408531 PMC2657552

[B57] Harmon-Jones E. Gable P. A. Peterson C. K. (2010). The role of asymmetric frontal cortical activity in emotion-related phenomena: a review and update. *Biol. Psychol.* 84 451–462. 10.1016/j.biopsycho.2009.08.010 19733618

[B58] Harrewijn A. Buzzell G. A. Debnath R. Leibenluft E. Pine D. S. Fox N. A. (2019). Frontal alpha asymmetry moderates the relations between behavioral inhibition and social-effect ERN. *Biol. Psychol.* 141 10–16. 10.1016/j.biopsycho.2018.12.014 30599209 PMC6471600

[B59] Harrewijn A. Schmidt L. A. Westenberg P. M. Tang A. van der Molen M. J. W. (2017). Electrocortical measures of information processing biases in social anxiety disorder: a review. *Biol. Psychol.* 129 324–348. 10.1016/j.biopsycho.2017.09.013 28964790

[B60] Harrewijn A. van der Molen M. J. W. van Vliet I. M. Houwing-Duistermaat J. J. Westenberg P. M. (2018a). Delta-beta correlation as a candidate endophenotype of social anxiety: a two-generation family study. *J. Affect. Disord.* 227 398–405. 10.1016/j.jad.2017.11.019 29154156

[B61] Harrewijn A. van der Molen M. J. W. van Vliet I. M. Tissier R. L. M. Westenberg P. M. (2018b). Behavioral and EEG responses to social evaluation: a two-generation family study on social anxiety. *Neuroimage* 17 549–562. 10.1016/j.nicl.2017.11.010 29527481 PMC5842666

[B62] Harrewijn A. Van der Molen M. J. W. Verkuil B. Sweijen S. W. Houwing-Duistermaat J. J. Westenberg P. M. (2018c). Heart rate variability as candidate endophenotype of social anxiety: a two-generation family study. *J. Affect. Disord.* 237 47–55. 10.1016/j.jad.2018.05.001 29763849

[B63] Harrewijn A. Van der Molen M. J. W. Westenberg P. M. (2016). Putative EEG measures of social anxiety: comparing frontal alpha asymmetry and delta-beta cross-frequency correlation. *Cogn. Affect. Behav. Neurosci.* 16 1086–1098. 10.3758/s13415-016-0455-y 27557885 PMC5153416

[B64] Herrmann C. S. Knight R. T. (2001). Mechanisms of human attention: event-related potentials and oscillations. *Neurosci. Biobehav. Rev.* 25 465–476. 11595268 10.1016/s0149-7634(01)00027-6

[B65] Hirsch C. R. Clark D. M. Mathews A. Williams R. (2003). Self-images play a causal role in social phobia. *Behav. Res. Ther.* 41 909–921. 12880646 10.1016/s0005-7967(02)00103-1

[B66] Hofmann S. G. (2000). Self-focused attention before and after treatment of social phobia. *Behav. Res. Ther.* 38 717–725. 10875193 10.1016/s0005-7967(99)00105-9

[B67] Hofmann S. G. (2007). Cognitive factors that maintain social anxiety disorder: a comprehensive model and its treatment implications. *Cogn. Behav. Ther.* 36 193–209. 18049945 10.1080/16506070701421313 PMC2151931

[B68] Iffland B. Sansen L. M. Catani C. Neuner F. (2014). The trauma of peer abuse: effects of relational peer victimization and social anxiety disorder on physiological and affective reactions to social exclusion. *Front. Psychiatry* 5:26. 10.3389/fpsyt.2014.00026 24672491 PMC3957367

[B69] Imperatori C. Farina B. Adenzato M. Valenti E. M. Murgia C. Marca G. D. (2019). Default mode network alterations in individuals with high-trait-anxiety: an EEG functional connectivity study. *J. Affect. Disord.* 246 611–618. 10.1016/j.jad.2018.12.071 30605880

[B70] Juruena M. F. Eror F. Cleare A. J. Young A. H. (2020). *The Role of Early Life Stress in HPA Axis and Anxiety. In Anxiety Disorders.* Berlin: Springer, 141–153. 10.1007/978-981-32-9705-0_9 32002927

[B71] Kandilarova S. Stoyanov D. Kostianev S. Specht K. (2018). Altered resting state effective connectivity of anterior insula in depression. *Front. Psychiatry* 9:83. 10.3389/fpsyt.2018.00083 29599728 PMC5862800

[B72] Kashdan T. B. Roberts J. E. (2007). Social anxiety, depressive symptoms, and post-event rumination: affective consequences and social contextual influences. *J. Anxiety Disord.* 21 284–301. 16859889 10.1016/j.janxdis.2006.05.009

[B73] Kawaguchi A. Nemoto K. Nakaaki S. Kawaguchi T. Kan H. Arai N. (2016). Insular volume reduction in patients with social anxiety disorder. *Front. Psychiatry* 7:3. 10.3389/fpsyt.2016.00003 26834652 PMC4720735

[B74] Kessler R. C. Chiu W. T. Demler O. Walters E. E. (2005). Prevalence, severity, and comorbidity of 12-month DSM-IV disorders in the national comorbidity survey replication. *Arch. Gen. Psychiatry* 62 617–627. 15939839 10.1001/archpsyc.62.6.617 PMC2847357

[B75] Kim S. (2012). Stress and EEG. *Commun. Comput. Inform. Sci.* 310 325–332. 10.1007/978-3-642-32692-9

[B76] Klawohn J. Meyer A. Weinberg A. Hajcak G. (2020). Methodological choices in event-related potential (ERP) research and their impact on internal consistency reliability and individual differences: an examination of the error-related negativity (ERN) and anxiety. *J. Abnorm. Psychol.* 129:29. 10.1037/abn0000458 31868385 PMC6931902

[B77] Klumpp H. Angstadt M. Phan K. L. (2012). Insula reactivity and connectivity to anterior cingulate cortex when processing threat in generalized social anxiety disorder. *Biol. Psychol.* 89 273–276. 10.1016/j.biopsycho.2011.10.010 22027088 PMC3260042

[B78] Knyazev G. G. (2007). Motivation, emotion, and their inhibitory control mirrored in brain oscillations. *Neurosci. Biobehav. Rev.* 31 377–395. 17145079 10.1016/j.neubiorev.2006.10.004

[B79] Knyazev G. G. (2012). EEG delta oscillations as a correlate of basic homeostatic and motivational processes. *Neurosci. Biobehav. Rev.* 36 677–695. 10.1016/j.neubiorev.2011.10.002 22020231

[B80] Knyazev G. G. Savostyanov A. N. Bocharov A. V. Aftanas L. I. (2019). EEG cross-frequency correlations as a marker of predisposition to affective disorders. *Heliyon* 5:e02942. 10.1016/j.heliyon.2019.e02942 31844779 PMC6895656

[B81] Knyazev G. G. Savostyanov A. N. Bocharov A. V. Tamozhnikov S. S. Kozlova E. A. Leto I. V. (2019). Cross-frequency coupling in developmental perspective. *Front. Hum. Neurosci.* 13:158. 10.3389/fnhum.2019.00158 31139068 PMC6527755

[B82] Knyazev G. G. Schutter D. J. L. G. van Honk J. (2006). Anxious apprehension increases coupling of delta and beta oscillations. *Int. J. Psychophysiol.* 61 283–287. 10.1016/j.ijpsycho.2005.12.003 16516317

[B83] Knyazev G. G. Slobodskaya H. R. (2003). Personality trait of behavioral inhibition is associated with oscillatory systems reciprocal relationships. *Int. J. Psychophysiol.* 48 247–261. 12798985 10.1016/s0167-8760(03)00072-2

[B84] Koivisto M. Revonsuo A. (2010). Event-related brain potential correlates of visual awareness. *Neurosci. Biobehav. Rev.* 34 922–934. 20005249 10.1016/j.neubiorev.2009.12.002

[B85] Kolassa I.-T. Kolassa S. Bergmann S. Lauche R. Dilger S. Miltner W. H. R. (2009). Interpretive bias in social phobia: an ERP study with morphed emotional schematic faces. *Cogn. Emot.* 23 69–95.

[B86] Kolassa I.-T. Kolassa S. Musial F. Miltner W. H. R. (2007). Event-related potentials to schematic faces in social phobia. *Cogn. Emot.* 21 1721–1744. 10.1080/17470919.2017.1304990 28276275

[B87] Kolassa I. T. Miltner W. H. R. (2006). Psychophysiological correlates of face processing in social phobia. *Brain Res.* 1118 130–141. 10.1016/j.brainres.2006.08.019 16970928

[B88] Langer J. K. Tonge N. A. Piccirillo M. Rodebaugh T. L. Thompson R. J. Gotlib I. H. (2019). Symptoms of social anxiety disorder and major depressive disorder: a network perspective. *J. Affect. Disord.* 243 531–538. 10.1016/j.jad.2018.09.078 30292147 PMC6202058

[B89] Lefevre A. Hurlemann R. Grinevich V. (2019). Imaging neuropeptide effects on human brain function. *Cell Tissue Res.* 375 279–286. 10.1007/s00441-018-2899-6 30069597

[B90] Liao W. Qiu C. Gentili C. Walter M. Pan Z. Ding J. (2010). Altered effective connectivity network of the amygdala in social anxiety disorder: a resting-state FMRI study. *PLoS One* 5:e15238. 10.1371/journal.pone.0015238 21203551 PMC3008679

[B91] Luck S. J. (2012). “Electrophysiological correlates of the focusing of attention within complex visual scenes: N2pc and related ERP components,” in *Oxford Library of Psychology. The Oxford Handbook of Event-Related Potential Components*, eds Luck S. J. Kappenman E. S. (Oxford: Oxford University Press), 329–360.

[B92] MacNamara A. Jackson T. B. Fitzgerald J. M. Hajcak G. Phan K. L. (2019). Working memory load and negative picture processing: neural and behavioral associations with panic, social anxiety, and positive affect. *Biol. Psychiatry* 4 151–159. 10.1016/j.bpsc.2018.04.005 29805056 PMC6197936

[B93] MacNamara A. Kappenman E. S. Black S. R. Bress J. N. Hajcak G. (2013). “Integrating behavioral and electrocortical measures of attentional bias toward threat,” in *Handbook of Self-Regulatory Processes in Development: New Directions and International Perspectives*, eds Barrett K. C. Fox N. A. Morgan G. A. Fidler D. J. Daunhauer L. A. (Washington, DC: American Psychological Association), 215–242.

[B94] Madison A. A. (2019). *Social Anxiety Symptoms, Heart Rate Variability, and Vocal Emotion Recognition: Evidence of a Normative Vagally-Mediated Positivity Bias in Women.* Ph.D. Thesis, The Ohio State University, Columbus, OH. 10.1080/10615806.2020.1839733 33156720

[B95] Makovac E. Meeten F. Watson D. R. Herman A. Garfinkel S. N. Critchley H. D. (2016). Alterations in amygdala-prefrontal functional connectivity account for excessive worry and autonomic dysregulation in generalized anxiety disorder. *Biol. Psychiatry* 80 786–795. 10.1016/j.biopsych.2015.10.013 26682467

[B96] Malenk R. C. Nestler E. J. Hyman S. E. (2009). Neural and neuroendocrine control of the internal milieu. *Mol. Neuropharmacol.* 246 248–259.

[B97] McNair N. A. Clapp W. C. Hamm J. P. Teyler T. J. Corballis M. C. Kirk I. J. (2006). Spatial frequency-specific potentiation of human visual-evoked potentials. *Neuroreport* 17 739–741. 16641679 10.1097/01.wnr.0000215775.53732.9f

[B98] Meiers G. Nooner K. Bellis De M. D. Debnath R. Tang A. (2020). Child abuse and neglect alpha EEG asymmetry, childhood maltreatment, and problem behaviors: a pilot home-based study. *Child Abuse Neglect.* 101:104358. 10.1016/j.chiabu.2020.104358 31958695 PMC7024668

[B99] Mellings T. M. B. Alden L. E. (2000). Cognitive processes in social anxiety: the effects of self-focus, rumination and anticipatory processing. *Behav. Res. Ther.* 38 243–257. 10665158 10.1016/s0005-7967(99)00040-6

[B100] Meynadasy M. Clancy K. Ke Z. Simon J. Wu W. Li W. (2019). Impaired early visual categorization of fear in social anxiety. *Psychophysiology* 57:e13509. 10.1111/psyp.13509 31788814 PMC7018552

[B101] Minkova L. Sladky R. Kranz G. S. Woletz M. Geissberger N. Kraus C. (2017). Task-dependent modulation of amygdala connectivity in social anxiety disorder. *Psychiatry Res. Neuroimaging* 262 39–46. 10.1016/j.pscychresns.2016.12.016 28226306

[B102] Miskovic V. Ashbaugh A. R. Santesso D. L. McCabe R. E. Antony M. M. Schmidt L. A. (2010). Frontal brain oscillations and social anxiety: a cross-frequency spectral analysis during baseline and speech anticipation. *Biol. Psychol.* 83 125–132. 10.1016/j.biopsycho.2009.11.010 19945500

[B103] Miskovic V. Campbell M. J. Santesso D. L. Van Ameringen M. Mancini C. L. Schmidt L. A. (2011a). Frontal brain oscillatory coupling in children of parents with social phobia: a pilot study. *J. Neuropsychiatry Clin. Neurosci.* 23 111–114. 10.1176/appi.neuropsych.23.1.111 21304147

[B104] Miskovic V. Moscovitch D. A. Santesso D. L. McCabe R. E. Antony M. M. Schmidt L. A. (2011b). Changes in EEG cross-frequency coupling during cognitive behavioral therapy for social anxiety disorder. *Psychol. Sci.* 22 507–516. 10.1177/0956797611400914 21378369

[B105] Miskovic V. Schmidt L. A. (2009). Frontal brain oscillatory coupling among men who vary in salivary testosterone levels. *Neurosci. Lett.* 464 239–242. 10.1016/j.neulet.2009.08.059 19716401

[B106] Miskovic V. Schmidt L. A. (2012). Social fearfulness in the human brain. *Neurosci. Biobehav. Rev.* 36 459–478. 21855571 10.1016/j.neubiorev.2011.08.002

[B107] Morillas-Romero A. Tortella-Feliu M. Bornas X. Putman P. (2015). Spontaneous EEG theta/beta ratio and delta–beta coupling in relation to attentional network functioning and self-reported attentional control. *Cogn. Affect. Behav. Neurosci.* 15 598–606. 10.3758/s13415-015-0351-x 25860658

[B108] Morrison A. S. Mateen M. A. Brozovich F. A. Zaki J. Goldin P. R. Heimberg R. G. (2019). Changes in empathy mediate the effects of cognitive-behavioral group therapy but not mindfulness-based stress reduction for social anxiety disorder. *Behav. Ther.* 50 1098–1111. 10.1016/j.beth.2019.05.005 31735245

[B109] Moscovitch D. A. Santesso D. L. Miskovic V. McCabe R. E. Antony M. M. Schmidt L. A. (2011). Frontal EEG asymmetry and symptom response to cognitive behavioral therapy in patients with social anxiety disorder. *Biol. Psychol.* 87 379–385. 10.1016/j.biopsycho.2011.04.009 21571033

[B110] Najjar R. Brooker R. J. (2017). Delta-beta coupling is associated with paternal caregiving behaviors during preschool. *Int. J. Psychophysiol.* 112 31–39. 10.1016/j.ijpsycho.2016.11.014 27884692 PMC5243183

[B111] Nathan P. E. (2019). “The DSM-IV process and outcomes,” in *Psychology, Science, And Human Affairs: Essays In Honor Of William Bevan*, eds Kessel F. Garmezy N. Trumbull R. Sokal M. (Abingdon: Routledge).

[B112] Newson J. J. Thiagarajan T. C. (2019). EEG frequency bands in psychiatric disorders: a review of resting state studies. *Front. Hum. Neurosci.* 12:521. 10.3389/fnhum.2018.00521 30687041 PMC6333694

[B113] Normann C. Schmitz D. Fürmaier A. Döing C. Bach M. (2007). Long-term plasticity of visually evoked potentials in humans is altered in major depression. *Biol. Psychiatry* 62 373–380. 10.1016/j.biopsych.2006.10.006 17240361

[B114] Peelen M. V. Atkinson A. P. Andersson F. Vuilleumier P. (2007). Emotional modulation of body-selective visual areas. *Soc. Cogn. Affect. Neurosci.* 2 274–283. 10.1093/scan/nsm023 18985133 PMC2566760

[B115] Perini S. J. Abbott M. J. Rapee R. M. (2006). Perception of performance as a mediator in the relationship between social anxiety and negative post-event rumination. *Cogn. Ther. Res.* 30 645–659.

[B116] Perna G. Alciati A. Sangiorgio E. Caldirola D. Nemeroff C. B. (2020). “Personalized clinical approaches to anxiety disorders,” in *Anxiety Disorders*, ed. Kim Y. K. (Berlin: Springer), 489–521. 10.1007/978-981-32-9705-0_25 32002943

[B117] Peschard V. Philippot P. Joassin F. Rossignol M. (2013). The impact of the stimulus features and task instructions on facial processing in social anxiety: an ERP investigation. *Biol. Psychol.* 93 88–96. 10.1016/j.biopsycho.2013.01.009 23384510

[B118] Phan K. L. Fitzgerald D. A. Nathan P. J. Tancer M. E. (2006). Association between amygdala hyperactivity to harsh faces and severity of social anxiety in generalized social phobia. *Biol. Psychiatry* 59 424–429. 16256956 10.1016/j.biopsych.2005.08.012

[B119] Phelps R. A. Brooker R. J. Buss K. A. (2016). Toddlers’ dysregulated fear predicts delta–beta coupling during preschool. *Dev. Cogn. Neurosci.* 17 28–34. 10.1016/j.dcn.2015.09.007 26624221 PMC4727986

[B120] Pittig A. Arch J. J. Lam C. W. R. Craske M. G. (2013). Heart rate and heart rate variability in panic, social anxiety, obsessive–compulsive, and generalized anxiety disorders at baseline and in response to relaxation and hyperventilation. *Int. J. Psychophysiol.* 87 19–27. 10.1016/j.ijpsycho.2012.10.012 23107994

[B121] Poole K. L. Anaya B. Pérez-edgar K. E. (2020). Behavioral inhibition and EEG delta-beta correlation in early childhood: comparing a between-subjects and within-subjects approach. *Biol. Psychol.* 149:107785. 10.1016/j.biopsycho.2019.107785 31628975 PMC6943939

[B122] Poole K. L. Schmidt L. A. (2019). Frontal brain delta-beta correlation, salivary cortisol, and social anxiety in children. *J. Child Psychol. Psychiatry* 60 646–654. 10.1111/jcpp.13016 30809809

[B123] Poppelaars E. S. Harrewijn A. Westenberg P. M. van der Molen M. J. W. (2018). Frontal delta-beta cross-frequency coupling in high and low social anxiety: an index of stress regulation? *Cogn. Affect. Behav. Neurosci.* 18 764–777. 10.3758/s13415-018-0603-7 29777479 PMC6096649

[B124] Potter D. R. (2019). Major depression disorder in adults: a review of antidepressants. *Int. J. Caring Sci.* 12:1936.

[B125] Prater K. E. Hosanagar A. Klumpp H. Angstadt M. Luan Phan K. (2013). Aberrant amygdala–frontal cortex connectivity during perception of fearful faces and at rest in generalized social anxiety disorder. *Depress. Anxiety* 30 234–241. 10.1002/da.22014 23184639 PMC3987867

[B126] Qiao J. Li A. Cao C. Wang Z. Sun J. Xu G. (2017). Aberrant functional network connectivity as a biomarker of generalized anxiety disorder. *Front. Hum. Neurosci.* 11:626. 10.3389/fnhum.2017.00626 29375339 PMC5770732

[B127] Qiu C. Liao W. Ding J. Feng Y. Zhu C. Nie X. (2011). Regional homogeneity changes in social anxiety disorder: a resting-state fMRI study. *Psychiatry Res.* 194 47–53. 10.1016/j.pscychresns.2011.01.010 21831605

[B128] Rachman S. Grüter-Andrew J. Shafran R. (2000). Post-event processing in social anxiety. *Behav. Res. Ther.* 38 611–617. 10846809 10.1016/s0005-7967(99)00089-3

[B129] Radtke S. R. Strege M. V. Ollendick T. H. (2020). “Exposure therapy for children and adolescents with social anxiety disorder,” in *Exposure Therapy for Children with Anxiety and OCD*, eds Peris T. S. Storch E. A. McGuire J. F. (Amsterdam: Elsevier), 193–219.

[B130] Rapee R. M. Heimberg R. G. (1997). A cognitive-behavioral model of anxiety in social phobia. *Behav. Res. Ther.* 35 741–756. 9256517 10.1016/s0005-7967(97)00022-3

[B131] Rapee R. M. Spence S. H. (2004). The etiology of social phobia: empirical evidence and an initial model. *Clin. Psychol. Rev.* 24 737–767. 15501555 10.1016/j.cpr.2004.06.004

[B132] Reyes N. Boulton K. A. Han J. Torok M. Wong Q. J. (2020). Cognitive bias modification for the induction of negative versus benign interpretations of the self in individuals with elevated social anxiety: effects on self-related and anxiety outcomes. *Cogn. Ther. Res.* 1–14.

[B133] Richards T. L. Grabowski T. J. Boord P. Yagle K. Askren M. Mestre Z. (2015). Contrasting brain patterns of writing-related DTI parameters, fMRI connectivity, and DTI–fMRI connectivity correlations in children with and without dysgraphia or dyslexia. *Neuroimage* 8 408–421. 10.1016/j.nicl.2015.03.018 26106566 PMC4473717

[B134] Rinck M. Telli S. Kampmann I. L. Woud M. L. Kerstholt M. Te Velthuis S. (2013). Training approach-avoidance of smiling faces affects emotional vulnerability in socially anxious individuals. *Front. Hum. Neurosci.* 7:481. 10.3389/fnhum.2013.00481 23970862 PMC3748377

[B135] Robichaud M. Koerner N. Dugas M. J. (2019). *Cognitive Behavioral Treatment for Generalized Anxiety Disorder: From Science to Practice.* Abingdon: Routledge.

[B136] Roh S. Kim J. S. Kim S. Kim Y. Lee S. (2020). Frontal alpha asymmetry moderated by suicidal ideation in patients with major depressive disorder: a comparison with healthy individuals. *Clin. Psychopharmacol. Neurosci.* 18 58–66. 10.9758/cpn.2020.18.1.58 31958906 PMC7006982

[B137] Sa L. (2005). *An introduction to the Event-Related Potential Technique.* Cambridge, MA: The MIT Press, 7–21.

[B138] Schmidt L. A. (2015). Frontal brain electrical activity in shyness and sociability. *Psychol. Sci.* 10 316–320.

[B139] Schreiber T. (2000). Measuring information transfer. *Phys. Rev. Lett.* 85 461. 10991308 10.1103/PhysRevLett.85.461

[B140] Schutter D. J. L. G. van Honk E. J. (2005). Salivary cortisol levels and the coupling of midfrontal delta-beta oscillations. *Int. J. Psychophysiol.* 55 127–129. 15598522 10.1016/j.ijpsycho.2004.07.003

[B141] Schutter D. J. L. G. van Honk J. (2004). Decoupling of midfrontal delta–beta oscillations after testosterone administration. *Int. J. Psychophysiol.* 53 71–73. 15172137 10.1016/j.ijpsycho.2003.12.012

[B142] Seok J.-W. Cheong C. (2019). Dynamic causal modeling of effective connectivity during anger experience in healthy young men: 7T magnetic resonance imaging study. *Adv. Cogn. Psychol.* 15 52–62. 10.5709/acp-0256-7 PMC7278524 32537036

[B143] Shahabi H. Moghimi S. (2016). Toward automatic detection of brain responses to emotional music through analysis of EEG effective connectivity. *Comput. Hum. Behav.* 58 231–239. 10.1016/j.chb.2016.01.005

[B144] Sladky R. Höflich A. Küblböck M. Kraus C. Baldinger P. Moser E. (2013). Disrupted effective connectivity between the amygdala and orbitofrontal cortex in social anxiety disorder during emotion discrimination revealed by dynamic causal modeling for fMRI. *Cereb. Cortex* 25 895–903. 10.1093/cercor/bht279 24108802 PMC4379995

[B145] Sokolov A. A. Zeidman P. Erb M. Ryvlin P. Pavlova M. A. Friston K. J. (2019). Linking structural and effective brain connectivity: structurally informed parametric empirical Bayes (si-PEB). *Brain Struct. Funct.* 224 205–217. 10.1007/s00429-018-1760-8 30302538 PMC6373362

[B146] Starcke K. Brand M. (2012). Decision making under stress: a selective review. *Neurosci. Biobehav. Rev.* 36 1228–1248. 10.1016/j.neubiorev.2012.02.003 22342781

[B147] Staugaard S. R. (2010). Threatening faces and social anxiety: a literature review. *Clin. Psychol. Rev.* 30 669–690. 10.1016/j.cpr.2010.05.001 20554362

[B148] Steptoe A. Hamer M. Chida Y. (2007). The effects of acute psychological stress on circulating inflammatory factors in humans: a review and meta-analysis. *Brain Behav. Immun.* 21 901–912. 17475444 10.1016/j.bbi.2007.03.011

[B149] Syal S. Hattingh C. J. Fouché J.-P. Spottiswoode B. Carey P. D. Lochner C. (2012). Grey matter abnormalities in social anxiety disorder: a pilot study. *Metab. Brain Dis.* 27 299–309. 10.1007/s11011-012-9299-5 22527992

[B150] Thibodeau R. Jorgensen R. S. Kim S. (2006). Depression, anxiety, and resting frontal EEG asymmetry: a meta-analytic review. *J. Abnorm. Psychol.* 115 715–729. 10.1037/0021-843X.115.4.715 17100529

[B151] Tononi G. Sporns O. (2003). Measuring information integration. *BMC Neuroscience* 4:31. 10.1186/1471-2202-4-31 14641936 PMC331407

[B152] Torrence R. D. Rojas D. C. Troup L. J. (2019). Awareness of emotional expressions in cannabis users: an event-related potential study. *Front. Psychol.* 10:69. 10.3389/fpsyg.2019.00069 30774608 PMC6367265

[B153] Valdes-Sosa P. A. Roebroeck A. Daunizeau J. Friston K. (2011). Effective connectivity: influence, causality and biophysical modeling. *Neuroimage* 58 339–361. 10.1016/j.neuroimage.2011.03.058 21477655 PMC3167373

[B154] van der Molen M. J. W. Dekkers L. M. S. Westenberg P. M. van der Veen F. M. van der Molen M. W. (2017). Why don’t you like me? Midfrontal theta power in response to unexpected peer rejection feedback. *Neuroimage* 146 474–483. 10.1016/j.neuroimage.2016.08.045 27566260

[B155] van Peer J. M. Roelofs K. Spinhoven P. (2008). Cortisol administration enhances the coupling of midfrontal delta and beta oscillations. *Int. J. Psychophysiol.* 67 144–150. 10.1016/j.ijpsycho.2007.11.001 18164501

[B156] Vassilopoulos S. P. Moberly N. J. Tsoumanis P. (2014). Social anxiety, anticipatory processing and negative expectancies for an interpersonal task in middle childhood. *J. Exp. Psychopathol.* 5 151–167.

[B157] Wang S.-Y. Lin I.-M. Fan S.-Y. Tsai Y.-C. Yen C.-F. Yeh Y.-C. (2019). The effects of alpha asymmetry and high-beta down-training neurofeedback for patients with the major depressive disorder and anxiety symptoms. *J. Affect. Disord.* 257 287–296. 10.1016/j.jad.2019.07.026 31302517

[B158] Wang W. Zhornitsky S. Li C. S.-P. Le T. M. Joormann J. Li C.-S. R. (2019). Social anxiety, posterior insula activation, and autonomic response during self-initiated action in a Cyberball game. *J. Affect. Disord.* 255 158–167. 10.1016/j.jad.2019.05.046 31153052 PMC6591038

[B159] Welander-Vatn A. Torvik F. A. Czajkowski N. Kendler K. S. Reichborn-Kjennerud T. Knudsen G. P. (2019). Relationships among avoidant personality disorder, social anxiety disorder, and normative personality traits: a twin study. *J. Personal. Disord.* 33 289–309. 10.1521/pedi_2018_32_341 29505386

[B160] Xhyheri B. Manfrini O. Mazzolini M. Pizzi C. Bugiardini R. (2012). Heart rate variability today. *Prog. Cardiovasc. Dis.* 55 321–331. 10.1016/j.pcad.2012.09.001 23217437

[B161] Xing M. Tadayonnejad R. MacNamara A. Ajilore O. DiGangi J. Phan K. L. (2017). Resting-state theta band connectivity and graph analysis in generalized social anxiety disorder. *Neuroimage* 13 24–32. 10.1016/j.nicl.2016.11.009 27920976 PMC5126152

[B162] Yeung R. C. Fernandes M. A. (2019). Altered working memory capacity for social threat words in high versus low social anxiety. *Anxiety Stress Coping* 32 505–521. 10.1080/10615806.2019.1626838 31232101

[B163] Yonkers K. A. Dyck I. R. Keller M. B. (2001). An eight-year longitudinal comparison of clinical course and characteristics of social phobia among men and women. *Psychiatr. Serv.* 52 637–643. 11331798 10.1176/appi.ps.52.5.637

[B164] Yuan M. Zhu H. Qiu C. Meng Y. Zhang Y. Shang J. (2016). Group cognitive behavioral therapy modulates the resting-state functional connectivity of amygdala-related network in patients with generalized social anxiety disorder. *BMC Psychiatry* 16:198. 10.1186/s12888-016-0904-8 27296506 PMC4906710

[B165] Yuan H. Zhu X. Tang W. Cai Y. Shi S. Luo Q. (2020). Connectivity between the anterior insula and dorsolateral prefrontal cortex links early symptom improvement to treatment response. *J. Affect. Disord.* 260 490–497. 10.1016/j.jad.2019.09.041 31539685

[B166] Zhang J. Hua Y. Xiu L. Oei T. P. Hu P. (2020). Resting state frontal alpha asymmetry predicts emotion regulation difficulties in impulse control. *Personal. Ind. Diff.* 159:109870.

[B167] Zhou Y. Friston K. J. Zeidman P. Chen J. Li S. (2017). The hierarchical organization of the default, dorsal attention and salience networks in adolescents and young adults. *Cereb. Cortex* 28 726–737. 10.1093/cercor/bhx307 29161362 PMC5929108

[B168] Ziegler M. G. (2012). “Psychological stress and the autonomic nervous system,” in *Primer on the Autonomic Nervous System*, eds Robertson D. Biaggioni I. Burnstock G. Low P. A. Paton J. F. R. (Amsterdam: Elsevier), 180–190.

